# Comparative Label-Free Proteomics Study on Celiac Disease-Active Epitopes in Common Wheat, Spelt, Durum Wheat, Emmer, and Einkorn

**DOI:** 10.1021/acs.jafc.4c02657

**Published:** 2024-06-21

**Authors:** Marie-Christin Norwig, Sabrina Geisslitz, Katharina A. Scherf

**Affiliations:** † Technical University of Munich, TUM School of Life Sciences, Freising 85354, Germany; ‡ Leibniz Institute for Food Systems Biology at the Technical University of Munich, Freising 85354, Germany; § Department of Bioactive and Functional Food Chemistry, Institute of Applied Biosciences, Karlsruhe Institute of Technology (KIT), Karlsruhe 76131, Germany; ∥ Technical University of Munich, TUM School of Life Sciences, Professorship of Food Biopolymer Systems, Freising 85354, Germany

**Keywords:** celiac disease, label-free quantitation, LC-MS/MS, multivariate data analysis, peptidomics

## Abstract

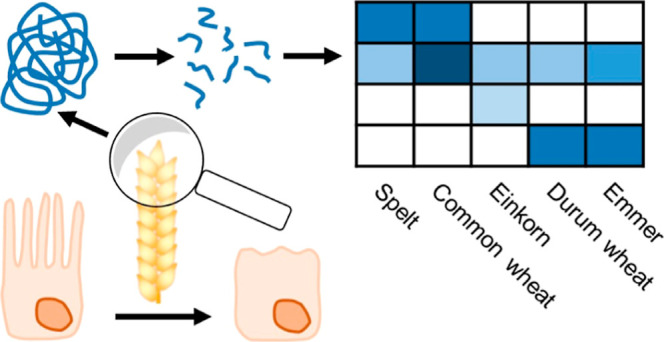

Wheat species with various ploidy levels may be different regarding their immunoreactive potential in celiac disease (CD), but a comprehensive comparison of peptide sequences with known epitopes is missing. Thus, we used an untargeted liquid chromatography tandem mass spectrometry method to analyze the content of peptides with CD-active epitope in the five wheat species common wheat, spelt, durum wheat, emmer, and einkorn. In total, 494 peptides with CD-active epitope were identified. Considering the average of the eight cultivars of each species, spelt contained the highest number of different peptides with CD-active epitope (193 ± 12, mean ± SD). Einkorn showed the smallest variability of peptides (63 ± 4) but higher amounts of certain peptides compared to the other species. The wheat species differ in the presence and distribution of CD-active epitopes; hence, the entirety of peptides with CD-active epitope is crucial for the assessment of their immunoreactive potential.

## Introduction

Celiac disease (CD) is a chronic immune-mediated inflammatory disease of the small intestine in genetically predisposed individuals triggered by dietary intake of storage proteins (gluten) from wheat, rye, or barley. The global prevalence of CD is estimated to be around 1%. The only therapy to avoid intestinal symptoms like diarrhea and extraintestinal disorders caused by malabsorption is a lifelong gluten-free diet.^1−4^ The pathomechanism of CD consists of cell damage in the small intestine caused by adaptive and innate immune response. Gluten peptides, either unmodified or deamidated by tissue transglutaminase, are recognized by the heterodimeric receptors HLA-DQ2 or HLA-DQ8 on the surface of antigen-presenting cells in the lamina propria. HLA-DQ2/DQ8 binds specific sequences consisting of nine amino acids, so-called epitopes. So far, 38 of them are known.^5^ The recognition of these epitopes provokes the adaptive immune reaction in CD patients, resulting in damage of the intestinal mucosa and apoptosis of epithelial cells.^6−8^

CD is not only triggered by the “modern” free-threshing wheat species common wheat (*Triticum aestivum* ssp. *aestivum*) and durum wheat (*T. turgidum* ssp. *durum*), but also by the “ancient” hulled wheat species spelt (*T. aestivum* ssp. *spelta*), emmer (*T. turgidum* ssp. *dicoccum*), and einkorn (*T. monococcum*). Regarding their genetic background, common wheat and spelt are hexaploid (AABBDD), durum wheat and emmer are tetraploid (AABB), and einkorn is diploid (A^m^A^m^).^1^ Despite the low production share of ancient wheat cultivars of less than 1% relative to total wheat production, they are of increasing interest concerning their immunoreactivity.

Based on the common wheat reference genome, immunoreactive proteins were mapped to the corresponding subgenomes and genes. Thereby, Juhász et al. identified many proteins with strong immunoreactivity in the D subgenome and fewer in the B subgenome.^9^ In accordance, Arora et al. found a lower expression of CD-active epitopes of α-gliadins in durum wheat than in common wheat, with genes on chromosome 6B showing the smallest expression.^10^ Differences in the genetic background of the species can also be detected by proteomic or peptidomics approaches. Prandi et al. analyzed four common wheat cultivars, three durum wheat cultivars, and one cultivar of einkorn, emmer, and spelt, each concerning their peptide distribution after simulated human digestion. They identified no significant differences in the amount of ten immunoreactive peptides among the species common wheat, durum wheat, and emmer, but there were significantly lower amounts in einkorn. Spelt showed significantly lower amounts than common wheat and durum wheat as well as lower amounts than emmer and higher amounts than einkorn.^11^ Asledottir et al. performed an ex vivo human digestion of porridge made of einkorn, emmer, spelt, or common wheat. When comparing the resulting peptide profiles, they detected more peptides with CD-active epitope in common wheat than in the other species.^12^ Malalgoda et al. detected 13 gliadin epitopes in 30 different common wheat cultivars. Additionally, they identified five epitopes in one einkorn sample and one epitope in one emmer sample.^13,14^ Based on the whole proteome, Afzal et al. recently compared the five species and identified a lower abundance of immunoreactive proteins in durum wheat and emmer compared to spelt and common wheat. Einkorn had the lowest abundance of immunoreactive proteins overall.^15^ Further, Geisslitz et al. analyzed the gluten content and composition of a large sample set including 15 cultivars each of the five wheat species. They found a significantly lower gluten and gliadin content in common wheat compared to the others and a higher glutenin content in common wheat and spelt than in durum wheat, emmer, and einkorn.^16^

These findings show that there are differences between the proteins of the wheat species and point to fewer immunoreactive sequences in tetraploid and especially diploid wheat species compared with hexaploid ones. To our knowledge, there are no comprehensive studies comparing the five wheat species focusing on peptides with CD-active epitope, to address the question of unequal abundance of CD-active epitopes and to demonstrate differences between species within distinct epitopes. Data covering all CD-active epitopes may allow the identification of wheat genotypes with a lower abundance of immunoreactive proteins. Additionally, more knowledge about the distribution of the epitopes in the species may be a good starting point for further research, e.g., wheat breeding or genetic modification with the aim to reduce immunoreactivity.

Therefore, the aim of this study was to investigate the differences in the content of peptides with at least one CD-active epitope in flours of common wheat, spelt, durum wheat, emmer, and einkorn using a comprehensive proteomics approach by untargeted label-free LC–MS/MS.

## Material and Methods

### Chemicals

All chemicals were of analytical or higher grade and purchased from VWR Merck (Darmstadt, Germany), Fisher Scientific (Waltham, USA), Carl Roth (Karlsruhe, Germany), AppliChem (Darmstadt, Germany), or Sigma-Aldrich (Steinheim, Germany).

### Samples

The sample set consisted of eight cultivars of each wheat species common wheat, spelt, durum wheat, emmer, and einkorn. The samples were cultivated by the State Plant Breeding Institute, University of Hohenheim (Germany) in Seligenstadt, Germany, and harvested in 2013. Longin et al. and Geisslitz et al. reported detailed information on the cultivars, the field trials, and the process of flour preparation.^17,18^ The cultivars and their abbreviations are summarized in Table S1.

### Gluten Extraction

Gluten extraction was performed after removal of the albumins/globulins.^19,20^ In brief, 150 mg of flour was extracted with 1.5 mL of phosphate-buffered saline (0.4 mol/L NaCl, 0.067 mol/L Na_2_HPO_4_/KH_2_PO_4_, pH 7.6) for 30 min at 22 °C, and the supernatant was removed after centrifugation (22 °C, 20 min, 3750*g*). For gluten extraction, the remaining residue was extracted twice with 1.5 mL of reducing extraction buffer [50% 1-propanol in water (v/v), 0.05 mol/L Tris–HCl, pH 8.5, 1% (w/v) dithiothreitol] for 30 min at 60 °C under nitrogen. The suspensions were centrifuged, and the supernatants were combined. Cysteine residues were alkylated with chloroacetamide (CAA) (600 μL, 0.5 mol/L CAA in 0.1 mol/L Tris–HCl, pH 8.5) for 45 min at 37 °C in the dark. The solvent was removed by evaporation to dryness. Three independent extractions were carried out for each flour sample.

### Enzymatic Digestion and Solid Phase Extraction

Tryptic and chymotryptic hydrolysis (1 mL, trypsin from bovine pancreas TPCK-treated, α-chymotrypsin from bovine pancreas TLCK-treated, enzyme-to-substrate ratio 1:50, 0.1 mol/L Tris–HCl, pH 7.8) was performed for 16 h at 37 °C in the dark.^20^ The reaction was stopped by heating the sample to 95 °C for 5 min. Samples were purified based on Lexhaller et al. by solid phase extraction using 500 mg Sep Pak tC18 cartridges (Waters, Eschborn, Germany).^21^ The C18-cartridges were activated with 3 mL of methanol, equilibrated with acetonitrile/water/formic acid (FA) (80:20:0.1; 3 mL) and washed with acetonitrile/water/FA (2:98:0.1; 5 × 3 mL). After the samples were loaded, the cartridges were washed again, and the peptides were eluted with acetonitrile/water/FA (40:60:0.1; 3 mL). The solvent was removed by evaporation to dryness, and the samples were reconstituted in 0.5 mL of 2% acetonitrile with 0.1% FA in water (v/v) and diluted 1:20 to an expected peptide concentration of 0.6 mg/mL.

### Liquid Chromatography Mass Spectrometry

An UltiMate 3000 RSLCnano system was coupled to a Q Exactive Plus Orbitrap mass spectrometer (Thermo Fisher Scientific, Waltham, MA, USA). Using a flow rate of 8 μL/min of 0.1% FA in water, the peptides were loaded onto a trap column for 5 min. Subsequently, the peptides were separated on an analytical column (Acclaim Pepmap C18 column, 2 μm, 75 μm × 150 mm, Thermo Fisher Scientific) using a flow rate of 300 nL/min with 0.1% FA in water (v/v) as solvent A, 0.1% FA and 5% water in acetonitrile as solvent B, and a gradient of 0–5 min 5% B, 5–60 min 5–40% B, 60–62 min 40–100% B, 62–65 min 100% B, 65–66 min 100–5% B, 66–80 min 5% B, and a column temperature of 40 °C. The eluate from the analytical column was sprayed via a Nanospray Flex Series ion source (Thermo Fisher Scientific) into the MS at a source voltage of 2.0 kV, at a capillary temperature of 250 °C and S-lens level of 60. The Q Exactive Plus was set to data-dependent acquisition in positive ion mode, automatically selecting the 30 most intense precursor ions from the preceding full MS1 spectrum with an isolation width of *m*/*z* 2.0 at 28% normalized collision energy and a default charge state of 2+. MS1 (*m*/*z* 360–1800) spectra were acquired in the Orbitrap using a resolution of 70,000 (at *m*/*z* 200), an automatic gain control (AGC) target of 3 × 10^6^ and a maximum injection time (IT) of 50 ms. MS2 spectra, selecting ions with charge 2+ to 7+, were acquired in the Orbitrap using a resolution of 17,500, an AGC target of 1 × 10^5^, a maximum IT of 50 ms, and a fixed first mass of *m*/*z* 140. Dynamic exclusion was set to 15 s. The injection volume was 0.5 μL. MS operation was performed with Xcalibur (Thermo Scientific, version 4.2.47).

### Peptide Identification

**Figure 1:**
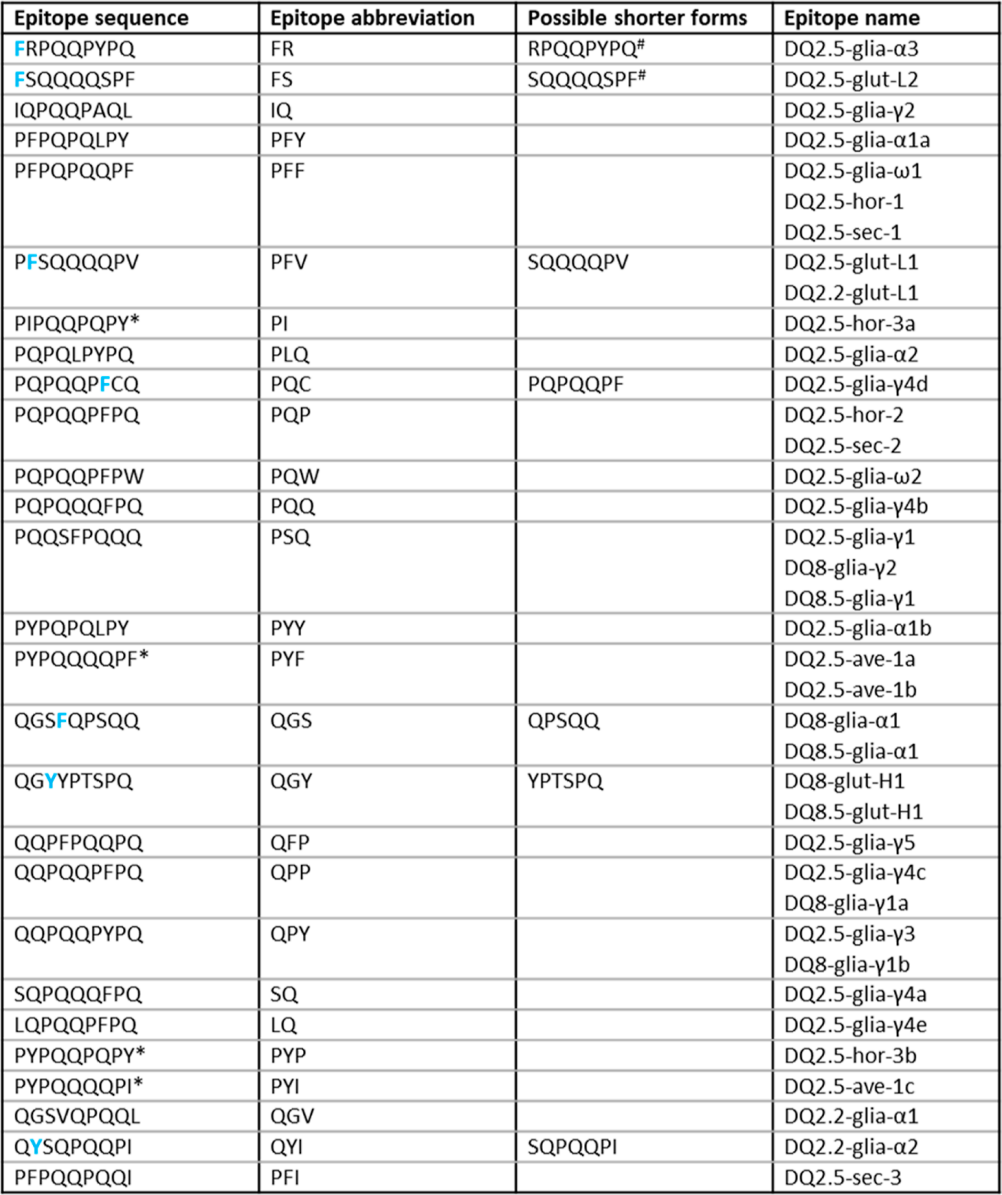
CD-active epitopes according to Sollid et al. (2019) after reversion of deamidation. The epitope names indicate the protein type in which the epitope is present: glia = gliadin, glut-L = low-molecular-weight glutenin subunits (LMW), glut-H = high-molecular-weight glutenin subunits (HMW), hor = hordein, sec = secalin, ave = avenin. (* not present in *Triticum* proteins, bold blue amino acids indicate possible cleavage sites of trypsin and chymotrypsin; # shorter forms that were included to the term “peptides with celiac disease active epitope”).

A database with all *Triticum* proteins with at least one CD-active epitope, called EProt-database, was generated as a basis for peptide identification (Table S2). Therefore, the *Triticum* proteome derived from UniProtKB (taxonomy: “Triticum [4564]”) was downloaded on 23.03.2021 and the sequences were filtered by the sequences of the CD-active epitopes with the help of a custom-built script. The sequences of epitopes present in wheat flour were identified after reversion of deamidation based on the known T-cell epitopes (Figure 1).^5^ Peptide identification was performed with the software MaxQuant (version 1.6.10.43)^22^ by searching the MS data against the EProt-database. Carbamidomethylation on cysteines was specified as fixed modification and chymotrypsin+ (cleavage sites: F, L, M, W, and Y) and trypsin (cleavage sites: K and R, but not before P) as proteolytic enzymes with up to ten allowed missed cleavage sites. The results were filtered for a minimal length of seven amino acids, a maximum peptide mass of 5000 Da, and 1% peptide and protein false discovery rate. Match between runs was activated, and the matching time window set to 0.7 min. Label-free quantitation (LFQ) intensities were calculated for relative peptide quantitation of the individual peptides across all samples.^23^ Other parameters were set as the default.

### Data Analysis and Statistics

Data analysis and statistics were performed with R [version 4.1.3 (2022-03-10)],^24^ using the tidyverse package (version 1.3.1) for data transformation.^25^ Peptides identified as potential contaminants or reverse contaminants were excluded. Peptide identifications with an Andromeda search score of less than 40 and an intensity equal to 0 were excluded. Peptides had to be identified in at least two of the three technical replicates. Means of the LFQ intensities were calculated for the replicates. Peptides were filtered to select peptides containing at least one CD-active epitope (EPeps). For the epitopes FR and FS also, peptides starting with the shorter form of the epitope were selected to consider potential cleavage sites (Figure 1). Principal component analysis (PCA) was performed with the R package FactoMineR (version 2.4)^26^ of the mean LFQ intensities after log-transformation (ln). Visualization as heatmaps was performed with the R package pheatmap (version 1.0.12)^27^ of the mean LFQ intensities after log-transformation (ln) and ward.D2 was used as the clustering method.^28,29^ The visualization of proteins or peptides present in one or more species was performed with R package ggVennDiagram (version 1.2.0).^30^ One-way analysis of variance (ANOVA) was performed with the aov function.^31^ Significant differences were calculated by means of Tukey with the HSD.test function of the agricolae package (version 1.3–5) using α = 0.05 and default parameters. The Bonferroni method was used for pairwise *t*-tests.^27,32,33^

## Results

### Identification of Peptides with CD-Active Epitope

The reversion of transglutaminase-mediated deamidation of gluten peptides^5^ led to 27 nonredundant epitope sequences (Figure 1). The abbreviations used for these epitope sequences are based on the first two letters of the amino acid sequence and are listed in the second column of Figure 1. Twenty-three of them are present in the protein sequences of *Triticum* (UniProtKB database). The four other epitopes (PI, PYF, PYP, and PYI) were not present in any *Triticum* protein sequence but were assigned to barley or oats and were not considered for this study.

The enzymes used for the sample preparation have specific cleavage sites. Trypsin cleaves after the amino acids arginine (R) and lysine (K), but not if proline (P) is in position P1′, with a few exceptions. Chymotrypsin cleaves after tyrosine (Y), tryptophan (W), and phenylalanine (F), but also not if proline (P) follows.^34^ As indicated in Figure 1 with bold blue letters, some of the CD-active epitopes contain a potential cleavage site. To also cover cleaved epitopes, the search for EPeps was extended to these shorter forms. Peptides containing cleaved epitopes were checked for being specific for the epitope by confirming that the corresponding protein had the complete epitope sequence. Only peptides with the shorter forms of the epitopes FR and FS that were specific for the corresponding epitopes were added to the EPeps.

In the whole sample set, 484 peptides with complete CD-active epitopes were identified. Additionally, there was one peptide starting with the shorter form of FS and nine peptides starting with the shorter form of FR. The peptide sequences with the epitopes are listed in Table S3. The epitopes PQC and PFI were present in the *Triticum* protein database but not identified in any of the samples. One reason could be the possible cleavage within the sequence of PQC. Additionally, the number of proteins containing these epitopes (22 proteins for PQC and 29 proteins for PFI) was low compared to, e.g., 737 for QGV. The epitope PFI was only assigned to rye and is likely not present in wheat. The MS data and MaxQuant result tables are available on ProteomeXchange with the data set identifier PXD050917.

**Figure 2:**
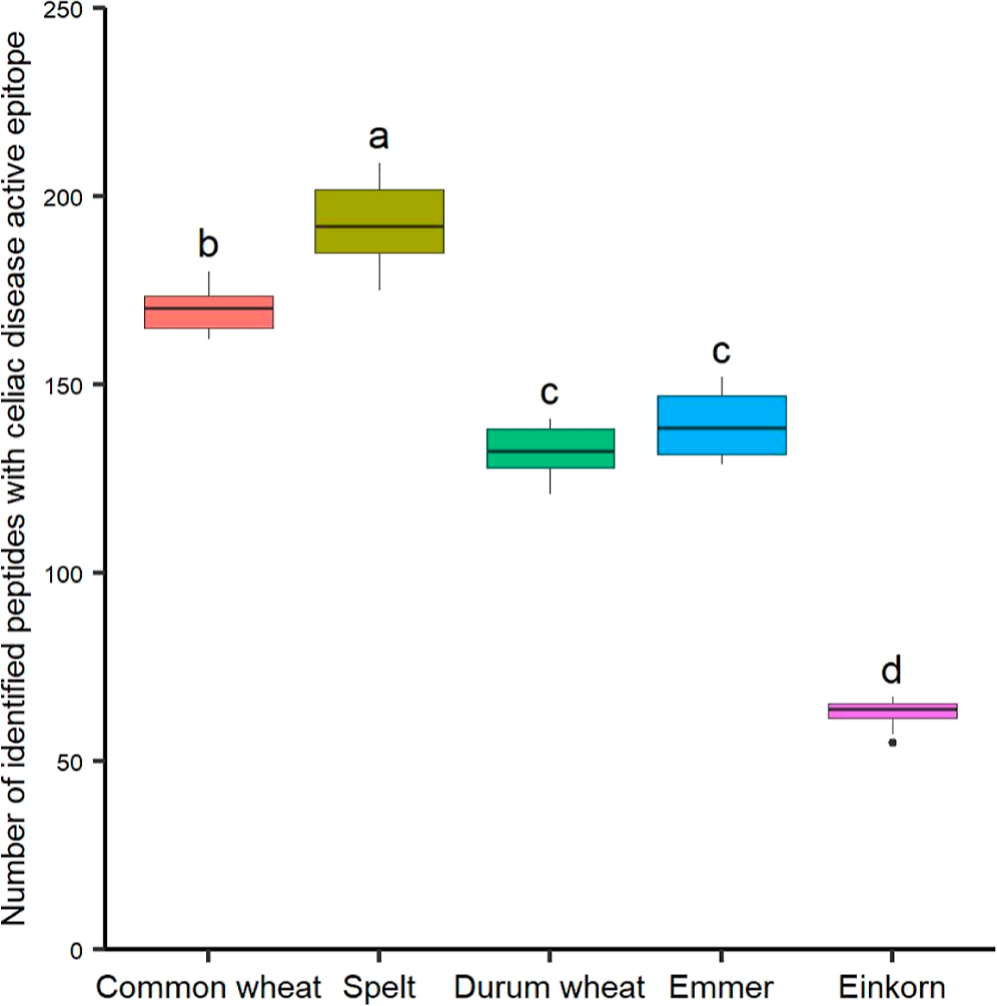
Number of identified peptides with CD-active epitope in the samples grouped by wheat species. Small letters designate significant differences between the species based on eight cultivars each (one-way ANOVA and Tukey test; α = 0.05).

The identified EPeps had lengths between eight and 46 amino acids (Figure S1) and the number of identified EPeps varied between samples, ranging from 55 to 209 different EPeps per sample (Figure 2). On average of the eight cultivars per species, spelt cultivars (193 ± 18) contained the highest number of different EPeps, followed by common wheat (170 ± 10), emmer (140 ± 12), durum wheat (132 ± 11), and einkorn (63 ± 8). These differences in the number of identified EPeps were significant in all cases (α < 0.05) besides the pair of durum wheat and emmer.

**Figure 3:**
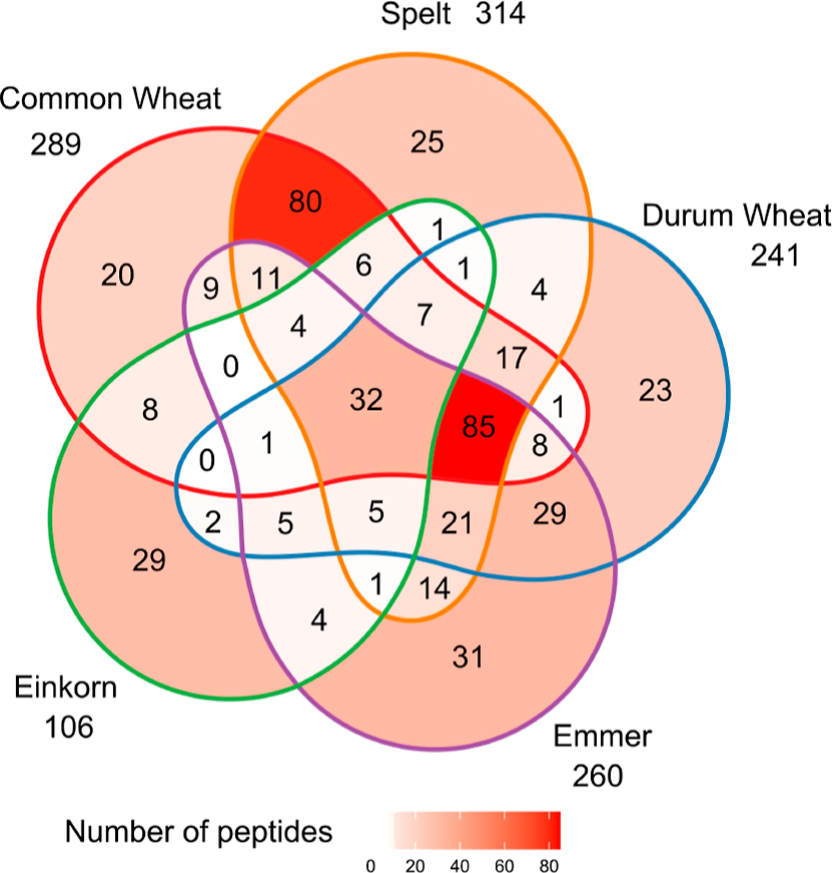
Number of peptides with CD-active epitope that are unique or common between the different wheat species common wheat, spelt, durum wheat, emmer, and einkorn, combining the results of eight cultivars of each species.

Considering the combined results of the eight cultivars per species, 289 different EPeps were identified in common wheat, 314 in spelt, 241 in durum wheat, 260 in emmer, and 106 in einkorn (Figure 3). Thirty-two of the identified EPeps were present in all species. When comparing different species, the major overlap of identified EPeps was between common wheat, spelt, durum wheat, and emmer (85 EPeps), followed by the overlap between the hexaploid species common wheat and spelt (80 EPeps). Emmer showed the highest number of EPeps specific for the species (31 EPeps), followed by einkorn (29 EPeps), spelt (25 EPeps), durum wheat (23 EPeps), and common wheat with 20 EPeps. Species-specific EPeps being present in all cultivars of a species existed only in einkorn (one EPep) and spelt (five EPeps). The number of cultivars per species, in which the EPeps were identified, is listed in Table S4.

According to the protein names in the UniProtKB database, the majority of the proteins corresponding to the identified EPeps belonged to gluten (1995 of 2113, i.e., 94.4%). The other 118 proteins are characterized as alpha-amylase inhibitor proteins (104 of 2113), “Progesterone binding protein (Fragment)” (1 of 2113), or are uncharacterized (13 of 2113). This reflects the protein database used (EProt) in which 95% of the proteins were gluten proteins.

### Comparison of Relative Quantities of Peptides with CD-Active Epitope

**Figure 4:**
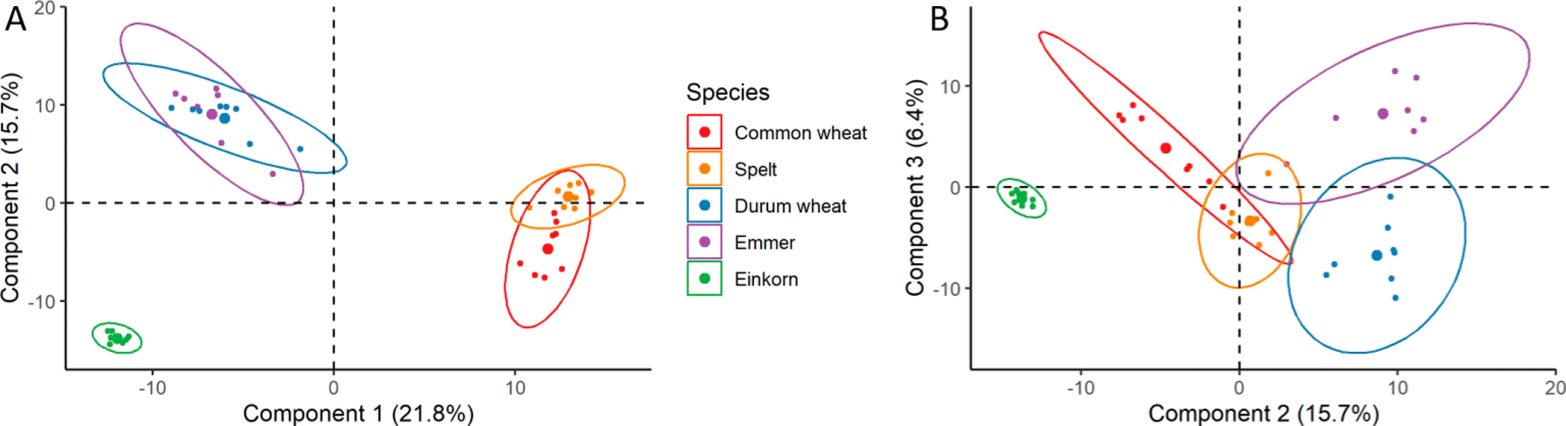
PCA of LFQ intensities of peptides with CD-active epitope showing components 1 and 2 (A) and components 2 and 3 (B).

The mean LFQ intensities of each EPep in triplicate of each cultivar were used for the assessment of relative quantities. The PCA of these mean LFQ intensities of the EPeps in all 40 samples enabled the differentiation between the species (Figure 4). Component 1 explained 21.8% of the overall variation, principal component 2 15.7% variation, and principal component 3 6.4% variation. The first component differentiated the hexaploid species from the tetra- and diploid species. The second component enabled the differentiation between the tetraploid species and diploid einkorn. The third component enabled differentiation within the tetra- and hexaploid species. The significances and statistical parameters of the PCA are shown in Tables S5 and S6.

### Cluster Analysis of Peptides with CD-Active Epitope

**Figure 5:**
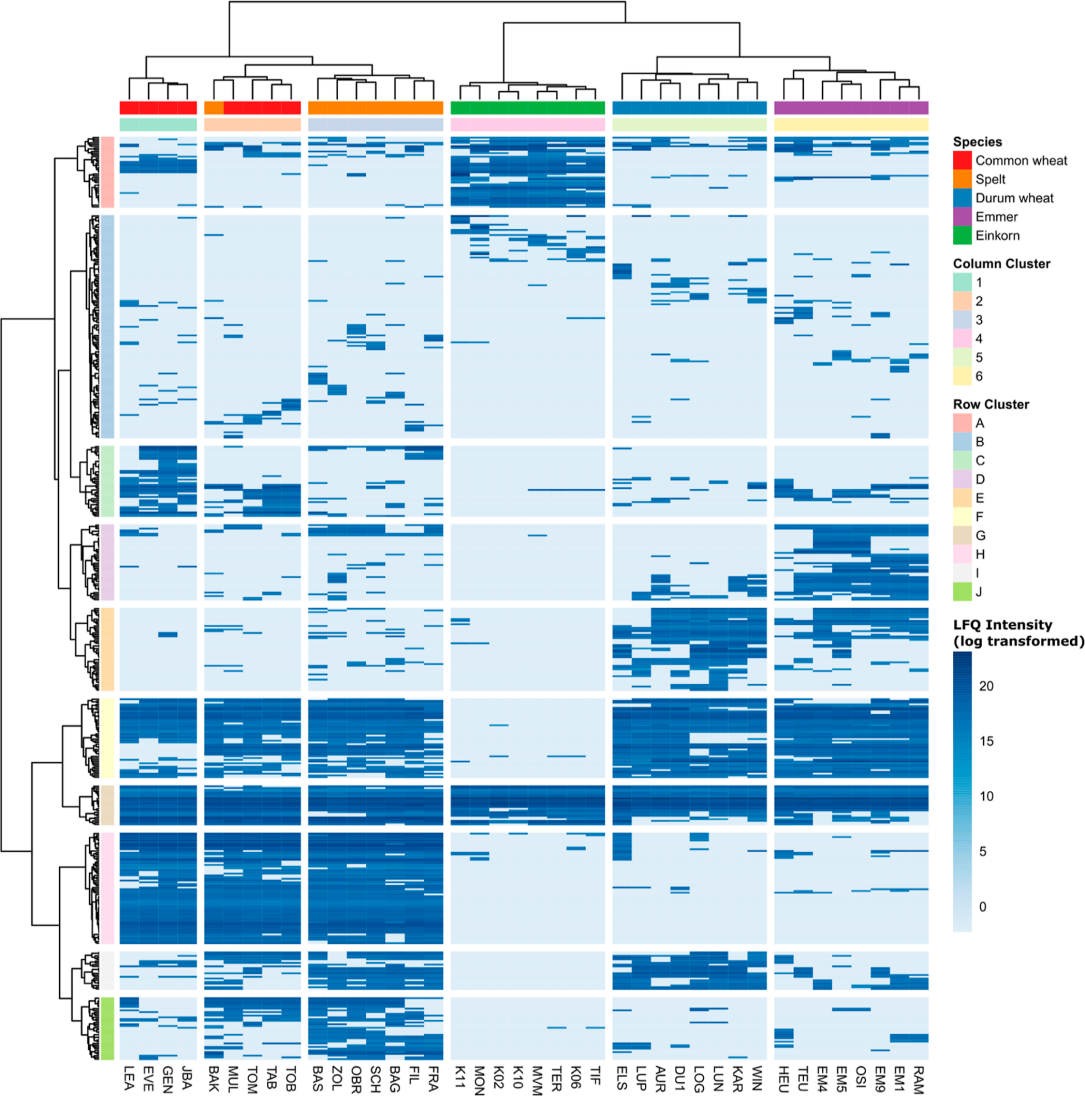
Intensity based on label-free quantitation of all 494 identified peptides with CD-active epitope in common wheat, spelt, durum wheat, emmer, and einkorn samples.

Ten EPeps were identified in all samples, and 171 EPeps in less than five samples. Figure 5 shows an overview of the mean LFQ intensities of all 494 identified EPeps after log-transformation. The dendrogram of the samples visualized that there was a major difference between the tetra- and diploid species versus the hexaploid species. In a second step, the samples of the tetra- and diploid species clustered perfectly by species (columns 4, 5, and 6). The hexaploid samples clustered in four common wheat samples (column cluster 1), seven spelt samples (column cluster 3), and a cluster of one spelt sample (BAK) and four common wheat samples (column cluster 2), illustrating that the common wheat samples MUL, TOM, TAB, and TOB were more similar to the spelt samples than to the other common wheat samples. Overall, the results illustrate the strong effect of the ploidy level on the proteome and also the presence of CD-active epitopes.

The EPeps clustered from the top to the bottom in EPeps mainly present in einkorn but also in samples of the other species (row cluster A), those present in some samples of all species (row cluster B), those mainly present in common wheat of column cluster 1 but also in all other species (row cluster C), EPeps present in the majority of emmer samples and some others (row cluster D), those present in the majority of durum wheat and emmer samples (row cluster E), those present in the majority of hexa- and tetraploid samples (row cluster F), those present in the majority of all samples (row cluster G), those mainly present in hexaploid samples (row cluster H), those mainly present in spelt and durum wheat, also in common wheat and emmer but not in einkorn (row cluster I) and EPeps present in the majority of spelt samples, some common wheat samples, as well as single tetraploid samples (row cluster J).

### Grouping of Peptides by CD-Active Epitope and Gluten Protein Type

Independently of row clusters A–J, the identified EPeps can be grouped by the epitope sequence and the gluten protein type to which the corresponding protein belongs. Gluten proteins can be divided by type into α-, γ-, and ω-gliadins as well as low- and high-molecular-weight glutenin subunits (LMW-GS and HMW-GS).^35^ The following highlights the differences between and within the wheat species based on the presence of CD-active epitopes. Additional descriptions of the EPeps in all gluten protein types are provided in the Supporting Information (Texts S1–S9).

### Peptides Derived from LMW-GS

**Figure 6:**
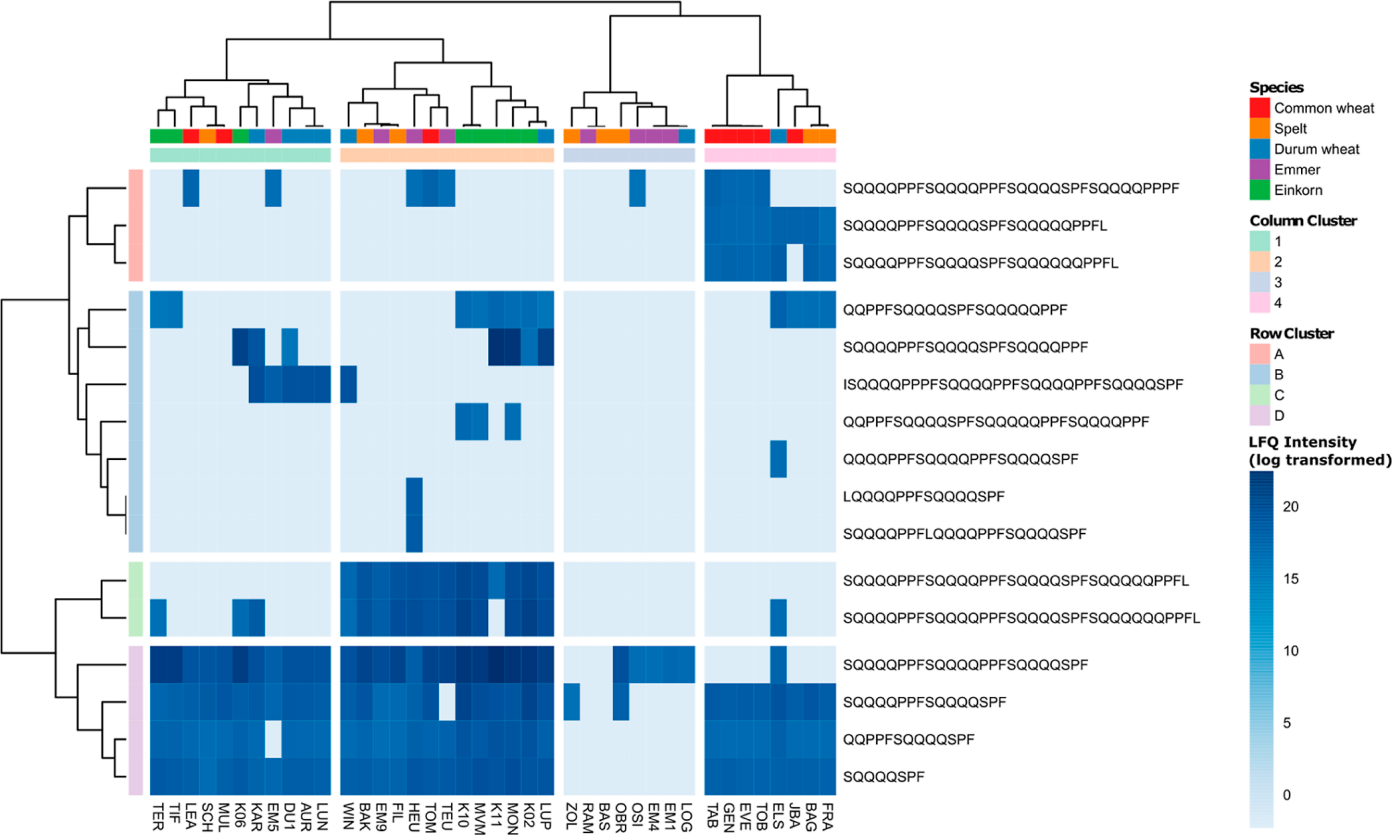
Intensity based on label-free quantitation of identified peptides with the CD-active epitope FSQQQQSPF belonging to proteins of the LMW-GS in common wheat, spelt, durum wheat, emmer, and einkorn samples.

LMW-GS contained either the epitope FS or PFV. Regarding the presence of EPeps with epitope FS (Figure 6 and Text S1), the samples showed no clusters by species or ploidy level. The samples RAM (emmer) and BAS (spelt) did not contain any of the EPeps. In contrast to the overall view (Figure 5), the number of identified EPeps with epitope FS was higher in einkorn samples or comparable to those of the other wheat species. The number of EPeps with epitope FS as a percentage of the number of identified EPeps per sample was even significantly higher in einkorn (8–15%) compared to the other species (0–7%).

**Figure 7:**
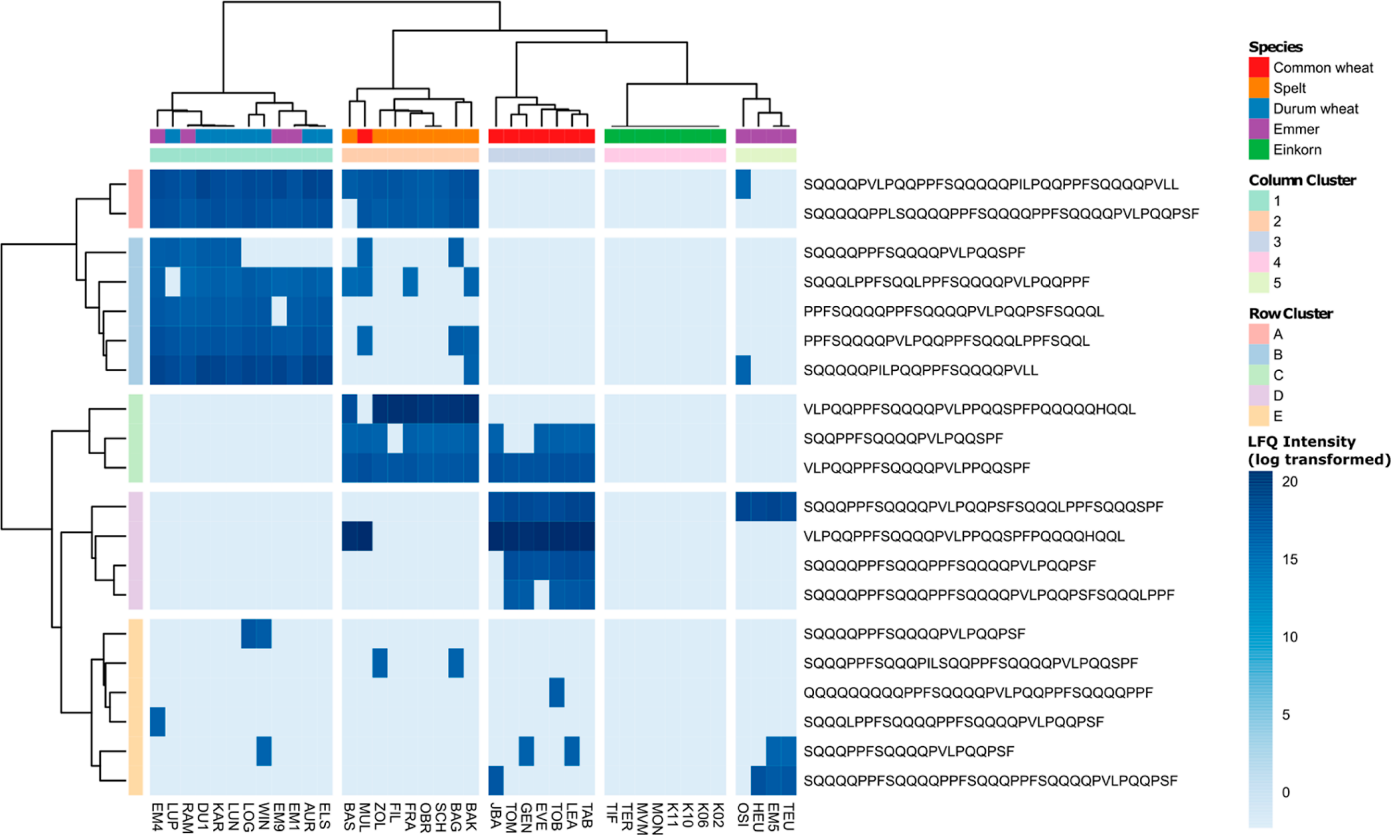
Intensity based on label-free quantitation of identified peptides with the CD-active epitope PFSQQQQPV belonging to proteins of the LMW-GS in common wheat, spelt, durum wheat, emmer, and einkorn samples.

The presence of EPeps with the epitope PFV in the samples enabled the differentiation of common wheat, spelt, and einkorn (Figure 7 and Text S2), with the exception of one common wheat cultivar (MUL) that was more similar to the spelt cultivars. Durum wheat and emmer were not separated from each other, while four of the emmer samples (column cluster 5) belonged to the main cluster of hexaploid and diploid samples (column cluster 2, 3, and 4) and were more similar to the einkorn samples (column cluster 4) than to the other tetraploid samples that were present in the second main cluster (column cluster 1). No EPeps with the epitope PFV were identified in einkorn samples. Concerning the presence of all epitopes of the LMW-GS, the variability within one species was higher than the differences between species or ploidy levels.

### Peptides Derived from HMW-GS

QGY is the only known epitope present in HMW-GS. The number of EPeps with the epitope QGY was high (80 EPeps; Figure S2 and Text S3). Based on the EPeps with epitope QGY, the samples clustered on the first level into three emmer samples (column cluster 1) and the einkorn samples (column cluster 2) as well as five clusters with the remaining samples. This second main cluster was separated into column cluster 3 with one emmer and six durum wheat samples, column cluster 4 with seven spelt and one common wheat sample, column cluster 5 with one spelt and four common wheat samples, column cluster 6 with three common wheat samples, and column cluster 7 with four emmer and two durum wheat samples. Each cluster contained only samples of the same ploidy level, but the dendrogram showed that the differentiation due to the ploidy level was not possible.

Interestingly, the resume of all glutenin EPeps (FS, PFV, and QGY) resulted in a differentiation of the species by the ploidy level. The separation by species was also possible, with the following exceptions: the spelt cultivar BAK belonged to the common wheat cluster and the durum wheat cultivars ELS and WIN to the emmer cluster (Figure S3).

### Peptides Derived from α-Gliadins

The epitopes FR, PYY, PFY, PLQ, QGS, QGV, and QYI are present in α-gliadins. Due to the overlap of the epitope sequences and the concurrent presence of several epitopes in one EPep, the α-gliadin epitopes were classified into epitope group 1 (QYI + FR), epitope group 2 (QGS + QGV), and epitope group 3 (PYY + PFY + PLQ).

Based on the presence of EPeps of epitope group 1 (Figure S4 and Text S4), the samples showed a separation by ploidy level. The hexaploid samples formed two clusters, of which one cluster contained four common wheat samples (column cluster 1) and the other contained the remaining hexaploid samples (column cluster 2). Additionally, one cluster contained the einkorn samples (column cluster 3) and one cluster contained the tetraploid species durum wheat and emmer (column cluster 4).

The number of EPeps with epitope group 2 was very high (127). The clustering of the species by the EPeps of epitope group 2 (Figure S5 and Text S5) was at first view comparable to that of epitope group 1. However, the dendrogram showed that four common wheat cultivars (column cluster 2) were more similar to the einkorn samples (column cluster 1) than the other samples including the remaining hexaploid samples (column cluster 3) and the tetraploid samples (column cluster 4). This means that there was no clustering by the ploidy level. The common wheat samples of column cluster 2 were the same as column cluster 1 of epitope group 1 (Figure S4), demonstrating differences in the α-gliadins with epitopes FR, QYI, QGS, and QGV within the common wheat samples.

The clustering of the species by the EPeps of epitope group 3 was similar to the one of epitope group 2 (Figure S6 and Text S6). Here, two durum wheat samples (column cluster 1) were more similar to the hexaploid samples (column cluster 2) than to the other tetraploid samples (column clusters 4 and 5) and the diploid samples (column cluster 3). EPep 4 of row cluster E (33-mer) is of particular interest as it contains all three epitopes (PYY, PFY, and PLQ) and was present not only in hexaploid samples but also in di- and tetraploid ones.

### Peptides Derived from γ- and ω-Gliadins

The epitope IQ and epitope group 4 (PSQ, SQ, and PQQ) were only present in γ-gliadins. The epitopes QPY, QPP, QFP, PFF, LQ, and PQW were present in γ- and ω-gliadins and were merged into epitope group 5. The grouping resulted due to the overlap of the epitope sequences and the concurrent presence of several epitopes in one EPep.

**Figure 8:**
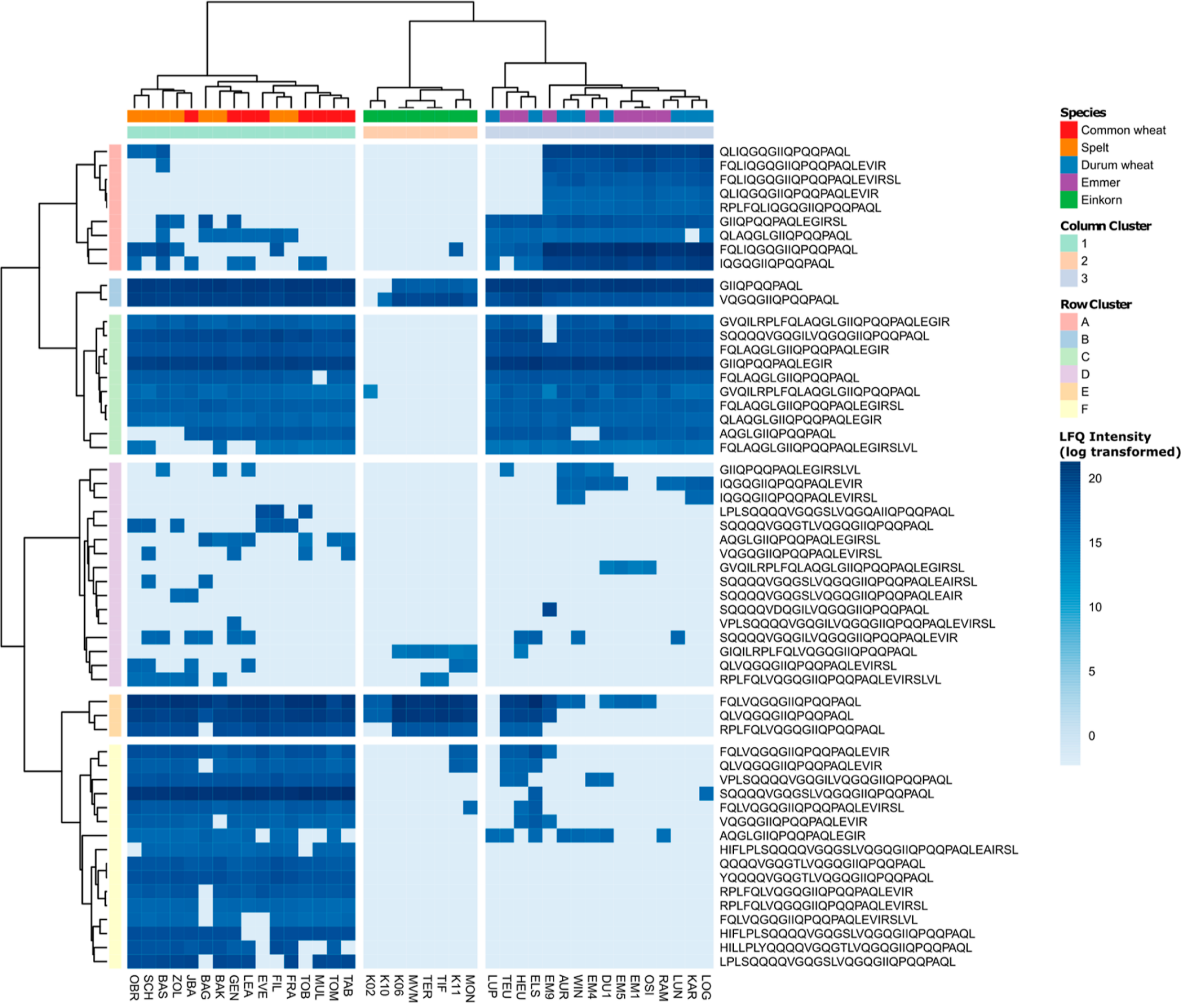
Intensity based on label-free quantitation of identified peptides with the CD-active epitope IQPQQPAQL belonging to γ-gliadin proteins in common wheat, spelt, durum wheat, emmer, and einkorn samples.

The samples clustered by EPeps with the epitope IQ according their ploidy level: hexaploid samples in column cluster 1, diploid samples in column cluster 2, and tetraploid samples in column cluster 3 (Figure 8 and Text S7). The dendrogram showed that there was the highest degree of similarity between di- and tetraploid samples.

Based on the presence of the EPeps of epitope group 4, the samples showed a separation of the hexaploid samples and the other ones (Figure S7 and Text S8). The hexaploid samples shared two clusters: the first one (column cluster 1) contained all spelt samples and one common wheat sample (MUL) and the second one (column cluster 2) contained the remaining common wheat samples. The diploid samples were present in column cluster 4. The tetraploid samples shared two clusters (column clusters 3 and 5), of which the samples of column cluster 5 were more similar to the einkorn samples (column cluster 4) than to the other tetraploid samples (column cluster 3).

The samples clustered consistently according to the species by the EPeps of epitope group 5 (column cluster 1 common wheat, column cluster 2 spelt, column cluster 3 einkorn, column cluster 4 emmer, and column cluster 5 durum wheat) (Figure S8 and Text S9). The number of EPeps with epitope group 5 was very high (112 EPeps).

### Quantitative Comparison of Peptides with CD-Active Epitope

**Figure 9:**
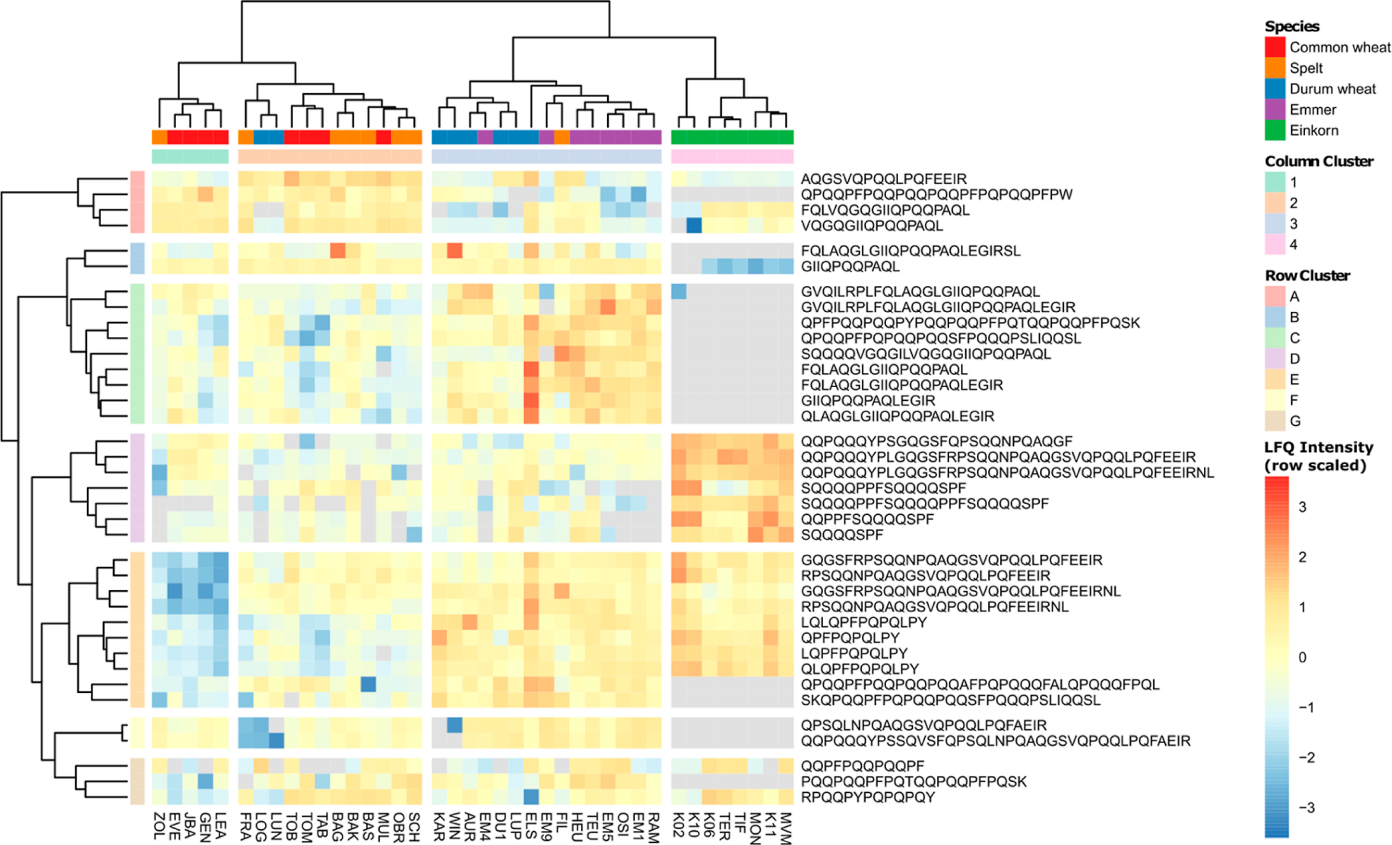
Scaled intensity based on label-free quantitation of peptides with CD-active epitope identified in more than 30 of 40 samples.

The quantitative comparison of EPeps identified in more than 30 out of 40 samples (Figure 9) showed a clustering of the samples according to their ploidy level, with some exceptions. Column clusters 1 and 2 contained hexaploid samples, and column cluster 2 additionally the two durum wheat samples LOG and LUN. Column cluster 3 contained tetraploid samples and the spelt sample FIL. Column cluster 4 contained all einkorn samples. Row cluster A contained EPeps with higher amounts in the hexaploid samples (column clusters 1 and 2) compared to tetra- and diploid samples (column clusters 3 and 4). EPeps of row cluster B were present in all hexa- and tetraploid samples and showed higher amounts in these samples compared to diploid samples. The EPeps of row cluster C showed the highest amounts in tetraploid samples (column cluster 3), especially in durum wheat sample ELS, and were not present in diploid samples, with one exception (first EPep in K02). Seven of nine EPeps in row cluster C contained the γ-gliadin epitope IQ. EPeps of row cluster D were present in all einkorn samples and showed higher amounts in einkorn compared to the other species. These EPeps contained the LMW-GS epitope FS or α-gliadin epitope QGS. EPeps of row cluster E showed lower amounts in hexaploid samples, especially the ones of column cluster 1 compared to other species. These EPeps contained the α-gliadin epitopes QGV or PFY or the γ-gliadin epitope QFP. EPeps of row cluster F were not present in einkorn samples and showed lower amounts in single samples [spelt FRA, durum wheat LOG, LUN, KAR (not present) and WIN] compared to the other samples. Differences in the amount of EPeps of row cluster G were not related to species or ploidy level.

## Discussion

We show comprehensive data of EPeps covering 21 CD-active epitopes in eight cultivars of each of five wheat species and demonstrate relevant differences between the species concerning their immunoreactive potential. To ensure comparability, the samples were all grown under the same conditions at one location in 1 year. One limitation is therefore that the environmental impact on the EPeps was not covered because at least three different locations or years would be needed.

We detected more than 490 EPeps covering 21 of 23 expected *Triticum* epitopes. Each sample contained between 55 and 209 EPeps. Spelt samples had the highest number of EPeps, as average of the eight cultivars, followed by common wheat, emmer, durum wheat, and einkorn. Asledottir et al. used a comparable LC–MS/MS method to analyze EPeps after ex vivo simulation of the human digestion and they found the lowest number of EPeps in einkorn, followed by spelt, emmer, and common wheat.^12^ The low number of EPeps in spelt, which is in contrast to our results, could be explained by different extraction and digestion protocols as well as differences within various cultivars of the same species.

The differentiation and overlap of the wheat species common wheat (AABBDD), spelt (AABBDD), durum wheat (AABB), emmer (AABB), and einkorn (A^m^A^m^) based on the EPeps followed the genetic relationship of the species.^36^ While 7% of the identified EPeps were present in all species, the major overlap was between common wheat, spelt, durum wheat, and emmer (18%), followed by the overlap between common wheat and spelt (17%). At the proteome level, Afzal et al. showed that most of the identified proteins were present in all species, followed by the overlap between common wheat, spelt, durum wheat, and emmer. Additionally, the differentiation of the five wheat species based on the proteome of 50 wheat cultivars was shown.^15^

In the present study, the distribution of EPeps was suitable to differentiate the wheat species, based on all EPeps as well as EPeps with the epitopes QPY, QPP, QFP, PFF, LQ, and PQW (epitope group 5). The EPeps with epitopes FR, QYI (epitope group 1), and IQ were more strongly affected by the ploidy level than by the species. EPeps containing the epitope FS did not follow the category species or ploidy level. Additionally, the second epitope of the LMW-GS PFV showed higher similarity within one species compared to FS, but the highest difference occurred between 12 tetraploid samples (column cluster 1) and the remaining samples covering all ploidy levels (Figure 6). Three emmer samples (EM4, EM5, and OSI) were more similar to the set of einkorn samples than to all the remaining samples based on their presence of the HMW-GS epitope QGY. Concluding, the variation of the epitopes of glutenins cannot be explained by the ploidy level or species. Reasons could be the low number of known glutenin epitopes (one for HMW-GS and two for LMW-GS)^5^ and that the glutenin proteins are encoded on all three subgenomes A, B, and D.^37,38^ The differences between the epitopes underline the importance of analyzing all epitopes to assess the immunoreactive potential instead of focusing on α-gliadins, as is often done.

EPeps that were present in all analyzed cultivars contained exclusively α-gliadin epitopes. Considering all epitopes, einkorn had a smaller number of different EPeps compared with the other species. In contrast, einkorn contained a higher number of EPeps with the epitope FS and higher amounts of some of the identified EPeps with epitopes FS, QGS, QGV, and PFY. No peptides with the epitopes PFV, SQ, PQQ, or QPY were identified in einkorn. No epitope was identified to be specific for a certain wheat species. Some cultivars did not contain certain epitopes, but no cultivar was identified with absence of all epitopes belonging to one HLA-DQ-type (Figure 1).

The quantitative comparison showed that not only is the presence of EPeps relevant to assess the CD-immunoreactive potential but also the amounts of the EPeps, in total and differentiated by epitope or gluten protein type. The higher amounts of EPeps with α-gliadin epitopes in einkorn go along with its higher gliadin content compared to the other species.^18^ Certain EPeps were already quantitated in wheat by other researchers. Prandi et al. analyzed the EPep RPQQPYPQPQPQ in four common wheat cultivars, three durum wheat cultivars and one einkorn, emmer and spelt cultivar each and found that einkorn contained a significantly higher amount than the other samples.^11^ This EPep is also part of the present study (Figure 6 last EPep) and showed the highest amounts in several einkorn samples (especially K06 and TER) and the hexaploid samples of column cluster 2.

Genomic analyses were used to map CD-active epitopes to the chromosomes and subgenomes of different wheat species. Several studies focused on CD-active epitopes on gliadin genes, resulting in partially contradictory assignments of the epitopes to the subgenes.^39−42^The epitopes PLQ and QGS were stated not to be encoded on the A subgenome^39^ but were also found on RNA belonging to the A subgenome of Indian wheat (PLQ and QGS)^40^ and spelt cultivars (only QGS).^41^ Salentijn et al. assigned the γ-gliadin epitopes IQ, PSQ, QPP, and QFP to all three subgenomes A, B, and D, whereas QPY was encoded on the D subgenome and PQQ on the B subgenome.^42^ By testing the recognition of gluten by T-cell clones specific for epitopes, Molberg et al. assigned epitopes to the subgenomes. The epitopes IQ, PSQ, SQ, and QFP were assigned to all three subgenomes A, B, and D, whereas PFY and QPY were assigned to the A and D subgenomes and PYY and PLQ only to the D subgenome.^43^ These findings were in contrast to the detection of peptides with PYY in tritordeum, which is a hybrid of durum wheat and a wild barley (AABBH^ch^H^ch^),^44^ and the identification of the epitope PLQ in durum wheat samples.^45^

The complete sequence of the highly immunogenic 33-mer with epitopes PYY, PFY, and PLQ overlapping was stated to be encoded on the D subgenome,^9^ and it was detected in small sample sets of hexaploid species but not in tetra- or diploid ones.^46,47^ The identification of EPeps with the α-gliadin epitopes PYY, PLQ, or QGS including the 33-mer in diploid or tetraploid samples is not in concordance with the genetic analyses because these species lack the D subgenome. Different parameters indicate the quality of the peptide identification including the kind of identification, number of identified precursor ions, number of MS/MS spectra, score, posterior error probability of the identification, signal intensity, and number of replicates, in which the peptide was identified. The quality of the identification of the 33-mer was somewhat less certain in the einkorn samples K02 and TER, all emmer samples (EM1, EM5, EM9, OSI, TEU), and the durum wheat sample WIN compared to the others but still fit the specified criteria. Further, the quality of the identification of the 33-mer in the einkorn samples K06 and TIF and the durum wheat samples ELS and LOG was comparable to that in hexaploid samples. Together with manual verification in the data set, we judged the identification of the 33-mer to be confident. Further investigations on the presence of the epitopes PYY, PLQ, and QGS in a larger number of cultivars of di- and tetraploid species combining genomic and proteomic analyses are therefore necessary. Such investigations can also be used to verify if certain peptides are indeed specific for one wheat species and could serve as marker peptides for authenticity testing.

For a valid interpretation of the results of this study, the following limitations have to be considered. The combination of trypsin and chymotrypsin was used for enzymatic hydrolysis. Trypsin is the most commonly used enzyme for proteomics analysis,^48^ but trypsin was not sufficient to generate the relevant EPeps due to the minimal presence of the amino acids R and K next to the epitopes. The additional use of chymotrypsin enables the generation of EPeps that cover 91% of EProts, but it leads to a higher number of missed cleavages and does not cover all possible EPeps. The simulation of the proteolysis during human digestion by the additional use of pepsin was avoided because pepsin is even less specific than chymotrypsin.^49,50^ Further, our aim was to comprehensively map EPeps in the flours and not to mimic human digestion. The MS analysis with data-dependent acquisition requires a protein database as the basis for the protein identification. Therefore, the quality of the protein database influences protein identification. The *Triticum* protein database used for this study and derived from UniProtKB in 2021 comprised an unequal distribution of proteins from the different species. Over 50% of the proteins were assigned to common wheat (*T. aestivum*), whereas emmer (*Triticum dicoccoides*) and einkorn (*Triticum monococcum*) accounted for less than 5% each. A more balanced protein database may lead to more peptide identifications, especially in emmer or einkorn, and could shift the ratio of EPeps between the species. Additionally, the epitope research is not completed yet, and the identification of further epitopes is expected. Additionally, the UniProtKB database continues to evolve and contains redundant, misnamed, or misaligned proteins. The EProt database used for this study contained 106 proteins named “AAI domain-containing proteins” (AAI = alpha-amylase inhibitor). These proteins were deleted (102) due to being duplicates or changed to protein names, indicating that they belong to the gliadin/glutenin family (4) in the UniProtKB database in the meantime. This means that no CD-active epitopes were actually found on alpha-amylase inhibitor protein sequences.

This study gives the first insight into the presence and distribution of CD-active epitopes in the different wheat species and demonstrates relevant differences between the species regarding their immunoreactive potential. However, the hypothesis of a lower immunoreactive potential of diploid or tetraploid wheat species compared to hexaploid ones cannot be confirmed or rejected due to high variability within the species and epitopes and unequal previous knowledge about the protein sequences of the species. Additionally, the CD immunoreactivity of cultivars and species with different distribution of CD-active epitopes has to be evaluated by in vitro assays or clinical studies to link the analytical data to immunologically relevant studies.

## Data Availability

The mass spectrometry proteomics data have been deposited to the ProteomeXchange Consortium via the PRIDE^51^ partner repository with the data set identifier PXD050917.

## Supporting Information Available

Overview of samples, statistical parameters of PCA of LFQ intensities, distribution of the number of identified peptides according to their length, additional descriptions of the figures, intensity based on label-free quantitation of identified peptides with CD-active epitopes QGYYPTSPQ, FSQQQQSPF, PFSQQQQPV, and QGYYPTSPQ belonging to HMW-GS, FRPQQPYPQ and QYSQPQQPI, QGSFQPSQQ and QGSVQPQQL, PYPQPQLPY, PFPQPQLPY, and PQPQLPYPQ belonging to α-gliadins; PQQSFPQQ, SQPQQQFPQ, and PQPQQQFPQ, and QQPQQPYPQ, QQPQQPFPQ, QQPFPQQPQ, PFPQPQQPF, LQPQQPFPQ, and PQPQQPFPW belonging to γ- and ω-gliadins in common wheat, spelt, durum wheat, emmer, and einkorn samples (PDF)
Protein database (XLSX)
Identified peptides with CD-active epitope, highlighting the epitope (XLSX)
Number of cultivars per species, in which the peptides with CD-active epitope were identified (XLSX)
