# Supplementary material for: Comparative Label-Free Proteomics Study on Celiac Disease-Active Epitopes in Common Wheat, Spelt, Durum Wheat, Emmer, and Einkorn
Source: J Agric Food Chem. 2024 Jun 21;72(26):15040–52. doi: 10.1021/acs.jafc.4c02657 (PMC11228976; doi:10.1021/acs.jafc.4c02657)
Supplement: Supplementary file 1 — jf4c02657_si_001.pdf [file jf4c02657_si_001.pdf]

Supporting Information

Comparative Label-Free Proteomics Study on Celiac Disease Active Epitopes in Common Wheat, Spelt, Durum Wheat, Emmer, and Einkorn

Marie-Christin Norwig[1,2], Sabrina Geisslitz[3], Katharina A. Scherf[2,3,4*]

[1] Technical University of Munich, TUM School of Life Sciences, Freising, 85354, Germany

[2] Leibniz Institute for Food Systems Biology at the Technical University of Munich, Freising, 85354, Germany

[3] Department of Bioactive and Functional Food Chemistry, Institute of Applied Biosciences, Karlsruhe Institute of Technology (KIT), Karlsruhe, 76131, Germany

[4] Technical University of Munich, TUM School of Life Sciences, Professorship of Food Biopolymer Systems, Freising, 85354, Germany

* Email: katharina.scherf@tum.de

Overview

Table S1. Overview of samples: Species, cultivar and abbreviation.

Table S2. Protein database: *Triticum* proteins (UniProtKB) with at least one celiac disease active epitope (according Sollid et al. 2019) as of 23.03.2021 (please see separate excel file).

Table S3. Identified peptides with celiac disease-active epitope highlighting the epitope (please see separate excel file).

Table S4. Number of cultivars per species, in which the peptides with celiac disease-active epitope were identified (please see separate excel file).

Table S5. Significance of components of principle component analysis of LFQ intensities of peptides with celiac disease active epitope based on the species.

Table S6. Statistical parameters of principle component analysis of LFQ intensities of peptides with celiac disease active epitope based on the species.

Figure S1. Distribution of the number of identified peptides with celiac disease active epitope according to their length.

Text S1. Additional description of Figure 6.

Text S2. Additional description of Figure 7.

Text S3. Additional description of Figure S2.

Figure S2. Intensity based on label-free quantitation of identified peptides with celiac disease active epitope QGYYPTSPQ belonging to proteins of the high-molecular-weight-glutenin subunits in common wheat, spelt, durum wheat, emmer and einkorn samples.

Figure S3. Intensity based on label-free quantitation of identified peptides with celiac disease active epitopes FSQQQQSPF, PFSQQQQPV and QGYYPTSPQ belonging to glutenin proteins in common wheat, spelt, durum wheat, emmer and einkorn samples.

Text S4. Additional description of Figure S4.

Figure S4. Intensity based on label-free quantitation of identified peptides with celiac disease active epitopes FRPQQPYPQ and QYSQPQQPI (epitope group 1) belonging to α-gliadin proteins in common wheat, spelt, durum wheat, emmer and einkorn samples.

Text S5. Additional description of Figure S5.

Figure S5. Intensity based on label-free quantitation of identified peptides with celiac disease active epitopes QGSFQPSQQ and QGSVQPQQL (epitope group 2) belonging to α-gliadin proteins in common wheat, spelt, durum wheat, emmer and einkorn samples.

Text S6. Additional description of Figure S6.

Figure S6. Intensity based on label-free quantitation of identified peptides with celiac disease active epitopes PYPQPQLPY, PFPQPQLPY and PQPQLPYPQ (epitope group 3) belonging to α-gliadin proteins in common wheat, spelt, durum wheat, emmer and einkorn samples.

Text S7. Additional description of Figure 8.

Text S8. Additional description of Figure S7.

Figure S7. Intensity based on label-free quantitation of identified peptides with celiac disease active epitopes PQQSFPQQ, SQPQQQFPQ and PQPQQQFPQ (epitope group 4) belonging to γ-gliadin proteins in common wheat, spelt, durum wheat, emmer and einkorn samples.

Text S9. Additional description of Figure S8.

Figure S8. Intensity based on label-free quantitation of identified peptides with celiac disease active epitopes QQPQQPYPQ, QQPQQPFPQ, QQPFPQQPQ, PFPQPQQPF, LQPQQPFPQ and PQPQQPFPW (epitope group 5) belonging to γ- and ω-gliadin proteins in common wheat, spelt, durum wheat, emmer and einkorn samples.

Table S1. Overview of samples: species, cultivar and abbreviation.

Cultivar	Abbreviation	Species
M-04018/01	K06	Einkorn
8.108/04	K02	Einkorn
M-04033/03	K10	Einkorn
M-07006/01	K11	Einkorn
Monlis	MON	Einkorn
MV Menket	MVM	Einkorn
Terzino	TER	Einkorn
Tifi	TIF	Einkorn
CC1E-04058/01	EM4	Emmer
CC1E-04059/01	EM5	Emmer
E-07087/02	EM9	Emmer
9.105/06/01	EM1	Emmer
Heulholzer Kolben/9.121/05	HEU	Emmer
Osiris	OSI	Emmer
Ramses	RAM	Emmer
Teutonia/9.131/05	TEU	Emmer
Auradur	AUR	Durum wheat
W-05005/02	DU1	Durum wheat
Elsadur	ELS	Durum wheat
Karur	KAR	Durum wheat
Logidur	LOG	Durum wheat
Lunadur	LUN	Durum wheat
Lupidur	LUP	Durum wheat
Wintergold	WIN	Durum wheat
Badengold	BAG	Spelt
Badenkrone	BAK	Spelt
Badenstern	BAS	Spelt
Filderstolz	FIL	Spelt
Franckenkorn	FRA	Spelt
Oberkulmer Rotkorn	OBR	Spelt
Schwabenkorn	SCH	Spelt
Zollernspelz	ZOL	Spelt
Event	EVE	Common wheat
Genius	GEN	Common wheat
JB Asano	JBA	Common wheat
Lear	LEA	Common wheat
Mulan	MUL	Common wheat
Tabasco	TAB	Common wheat
Tobak	TOB	Common wheat
Tommi	TOM	Common wheat

Table S2. Protein database: *Triticum* proteins (UniProtKB) with at least one celiac disease active epitope (according Sollid et al. 2019) as of 23.03.2021 (please see separate excel file).

Table S3. Identified peptides with celiac disease active epitope highlighting the containing epitope (please see separate excel file).

Table S4. Number of cultivars per species, in which the peptides with celiac disease active epitope were identified (please see separate excel file).

Table S5. Significance of components of principle component analysis of LFQ intensities of peptides with celiac disease active epitope based on the species.

Component	Coefficient of determination R2	p-value
1	0.985	2.302e-31
2	0.954	8.028e-23
3	0.794	1.420e-11

Table S6. Statistical parameters of principle component analysis of LFQ intensities of peptides with celiac disease active epitope based on the species.

Component	Species	Estimate	p-value
1	Spelt	12.919	1.826e-05
1	Common wheat	11.802	1.307e-04
1	Durum wheat	-6.024	6.944e-02
1	Emmer	-6.726	4.150e-02
1	Einkorn	-11.972	9.901e-05
2	Emmer	9.065	6.709e-04
2	Durum wheat	8.688	1.207e-03
2	Common wheat	-4.660	9.872e-02
2	Einkorn	-13.761	2.563e-09
3	Emmer	7.272	6.497e-06
3	Common wheat	3.871	2.959e-02
3	Spelt	-3.308	6.530e-02
3	Durum wheat	-6.746	4.262e-05

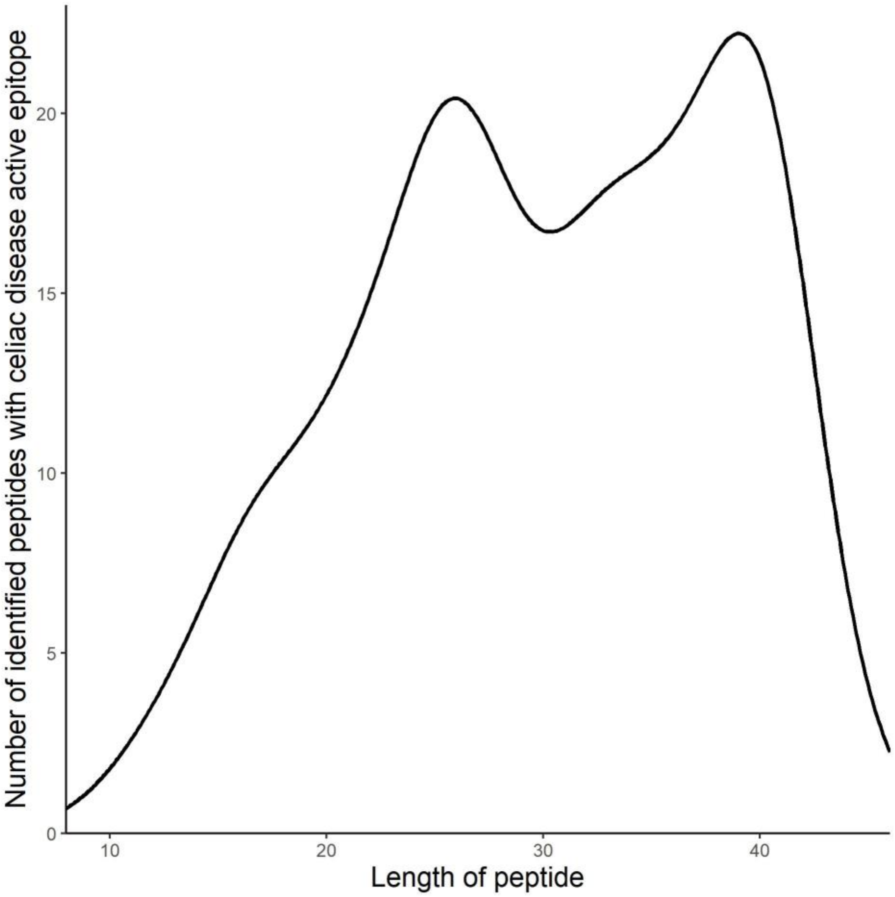

Figure S1. Distribution of the number of identified peptides with celiac disease active epitope according to their length between eight and 46 amino acids.

Text S1. Additional description of Figure 6.

Regarding the presence of EPeps with epitope FS (Figure 6), the samples showed no cluster by species or ploidy level. The samples RAM (emmer) and BAS (spelt) did not contain any of the EPeps. Column cluster 1 and 2 contained samples of all species whereas column cluster 3 only contained spelt, durum wheat and emmer samples and column cluster 4 contained the majority of common wheat samples, two spelt samples and one durum wheat sample. Row cluster A contained EPeps mainly present in samples of column cluster 4. EPeps of row cluster B were present in single samples but not in samples of column cluster 3. EPeps of row cluster C were present in all samples of column cluster 2 and in single samples of column cluster 1 and 4. Row cluster D contained EPeps present in the majority of samples in column cluster 1, 2 and 3.

Text S2. Additional description of Figure 7.

Row cluster A summarized EPeps present in the cluster of durum wheat and emmer (column cluster 1) and the cluster of spelt (column cluster 2). EPeps of row cluster B were present in the majority of the samples of column cluster 1 and single samples of column cluster 2 (spelt samples and common wheat sample MUL). In both row clusters, one EPep each was identified additionally in the emmer sample OSI. EPeps of row cluster C were present in the majority of hexaploid samples, but not in tetraploid and diploid samples. EPeps of row cluster D were present in the majority of common wheat samples. One EPep was additionally present in the spelt sample BAS and another EPep in the emmer samples of column cluster 5. Row cluster E summarized EPeps present in single samples.

Text S3. Additional description of Figure S2.

EPeps that were present in the majority of the einkorn samples were grouped in row cluster A. Only one EPep of this cluster was also present in some hexaploid samples. Row cluster B contained EPeps present in hexaploid samples and single tetraploid samples. The EPeps of row cluster C were present in samples of all species. Row cluster D contained EPeps present in all emmer samples of column cluster 1, and in one emmer sample of column clusters 3 and 7, respectively. Row cluster E summarized EPeps present in single samples of all species. The EPeps of row cluster F were present in the majority of the samples of column cluster 3 and in some samples of column cluster 4 and 7. Row cluster G contained EPeps present in all samples of column cluster 3 and 4 as well as in some samples of column cluster 5, 6 and 7. Row cluster H showed EPeps present in the hexaploid samples besides the ones of column cluster 6. The EPeps of row cluster I were present in all species except einkorn.

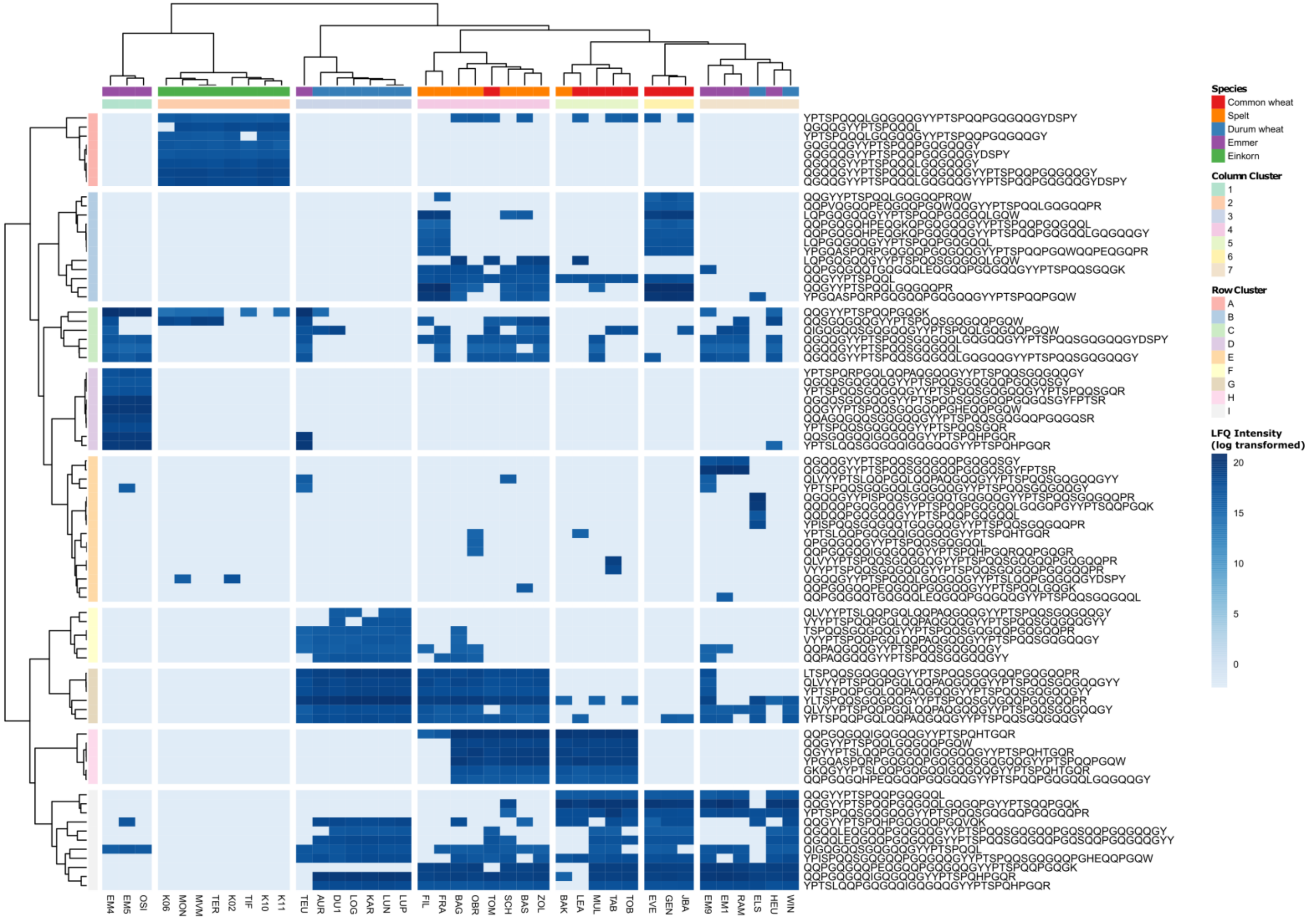

Figure S2. Intensity based on label-free quantitation of identified peptides with celiac disease active epitope QGYYPTSPQ belonging to proteins of the high-molecular-weight-glutenin subunits in common wheat, spelt, durum wheat, emmer and einkorn samples.

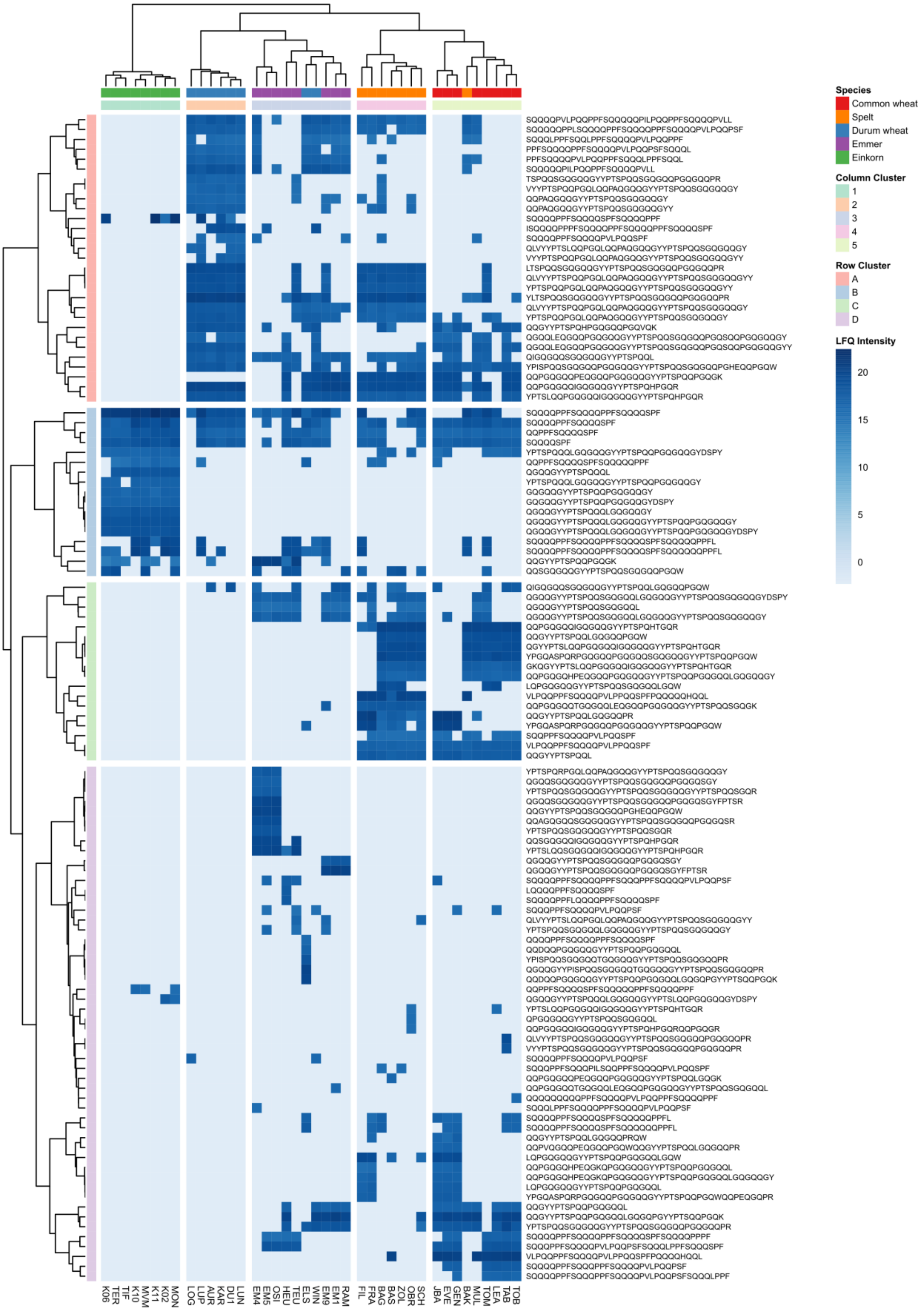

Figure S3. Intensity based on label-free quantitation of identified peptides with celiac disease active epitopes FSQQQQSPF, PFSQQQQPV and QGYYPTSPQ belonging to glutenin proteins in common wheat, spelt, durum wheat, emmer and einkorn samples.

Text S4. Additional description of Figure S4.

The EPep of row cluster A was present in all samples. Row cluster B contained EPeps mainly present in the hexaploid samples and in some tetraploid samples. The EPeps of row cluster C were mainly present in einkorn samples, but also in the other species. The EPeps of row cluster D were present in some tetraploid samples and single hexaploid samples. The EPeps of row cluster E showed a major variation of their presence in the different wheat species. Some of the EPeps of row cluster E were mainly present in the common wheat samples of column cluster 1. In contrast, others were not present in these common wheat samples but instead in single other hexa- or tetraploid samples.

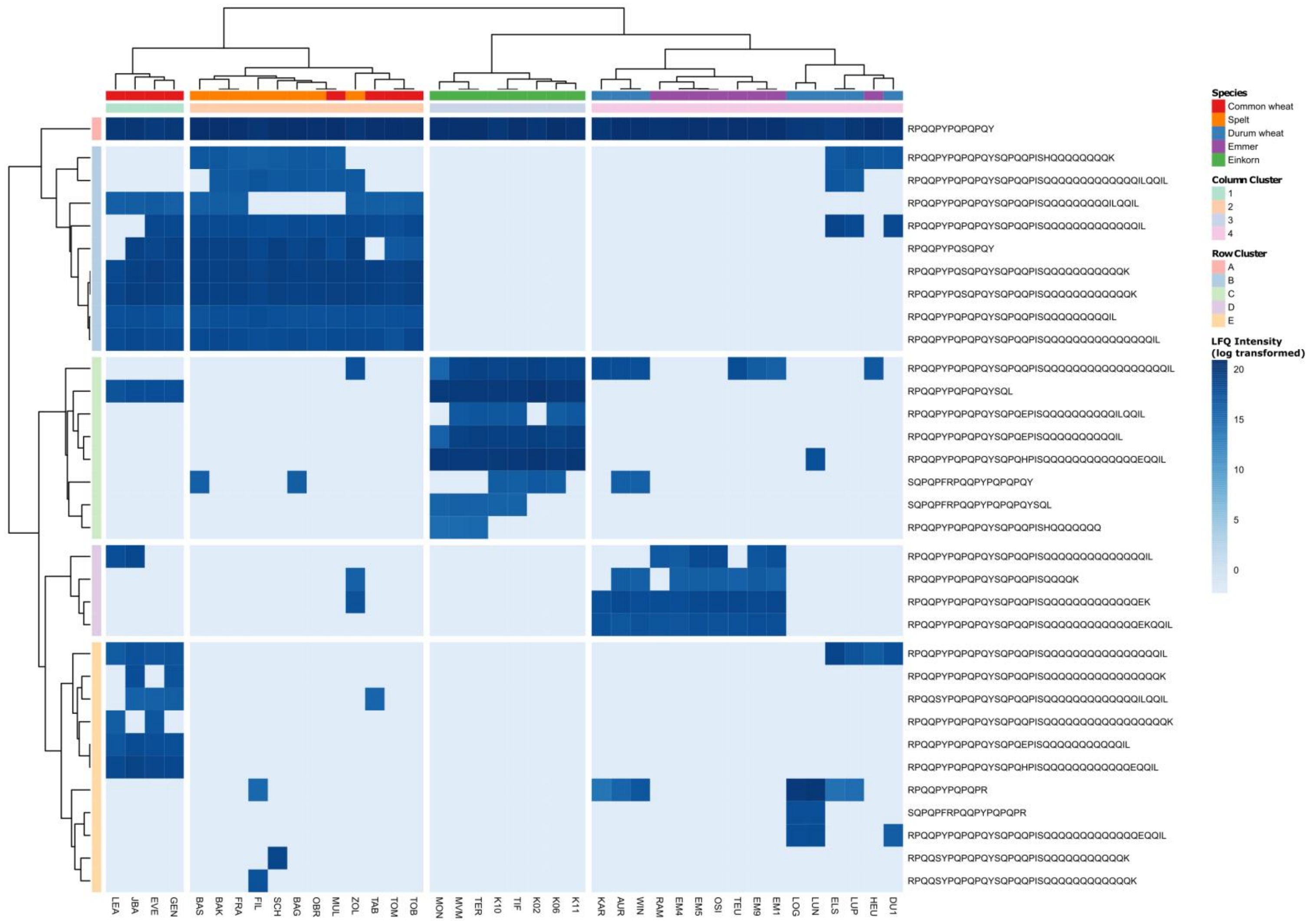

Figure S4. Intensity based on label-free quantitation of identified peptides with celiac disease active epitopes FRPQQPYPQ and QYSQPQQPI (epitope group 1) belonging to α-gliadin proteins in common wheat, spelt, durum wheat, emmer and einkorn samples.

Text S5. Additional description of Figure S5.

Row cluster A contained EPeps present in all samples. The EPeps of row cluster B were present in the majority of hexaploid and tetraploid samples. The EPeps of row cluster C were mainly present in hexaploid samples, the ones of row cluster D mainly in tetraploid samples. The EPeps of both clusters were also present in some samples of the other species. Row cluster E contained EPeps present in the einkorn samples and in the hexaploid samples of column cluster 2 with one exception of a spelt cultivar of column cluster 3 (BAS). Row cluster F summed up EPeps present in single samples over all wheat species. The second EPep of cluster A and the first EPep of cluster E contained the epitope QGS and were present in all or seven einkorn samples, respectively.

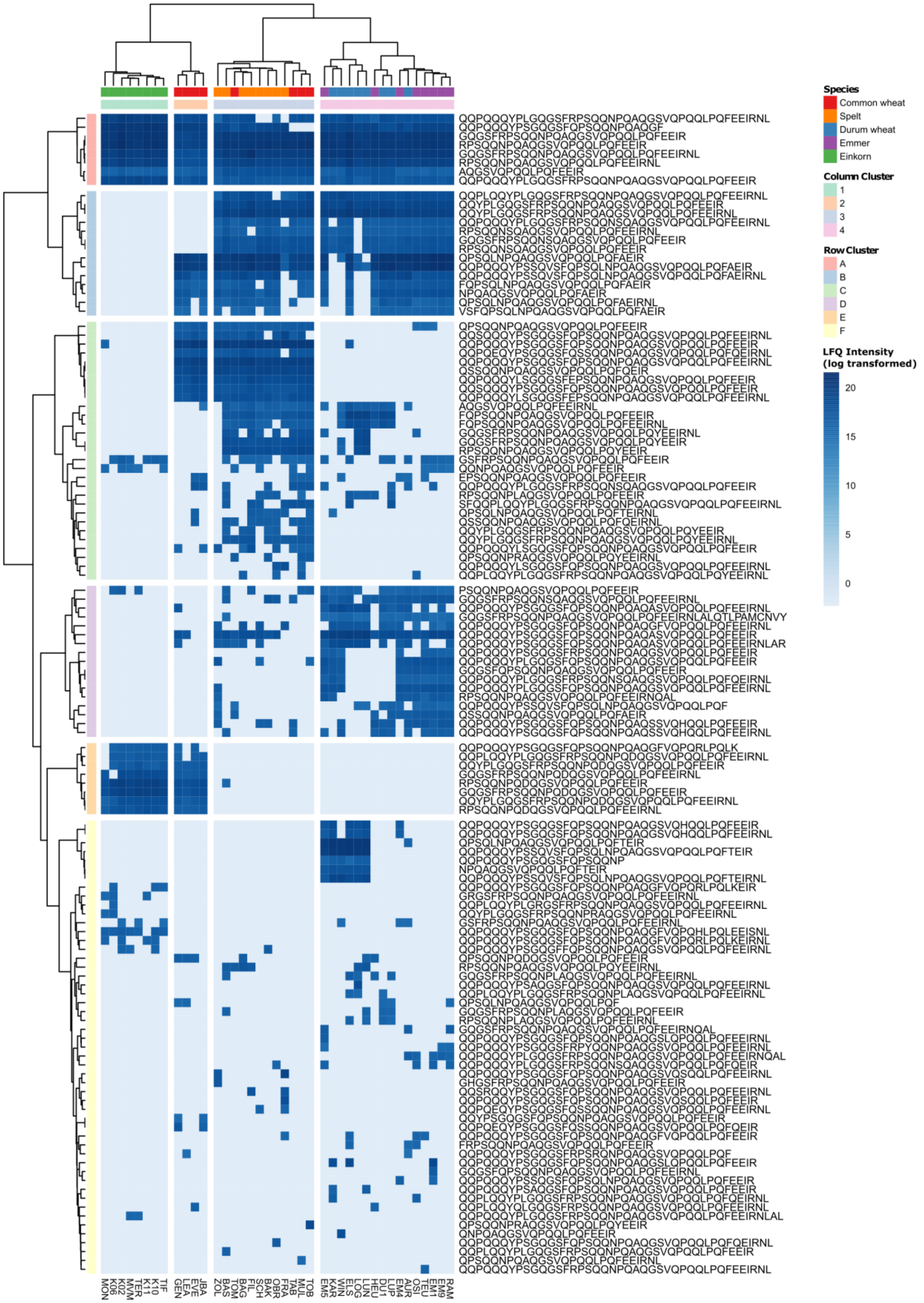

Figure S5. Intensity based on label-free quantitation of identified peptides with celiac disease active epitopes QGSFQPSQQ and QGSVQPQQL (epitope group 2) belonging to α-gliadin proteins in common wheat, spelt, durum wheat, emmer and einkorn samples.

Text S6. Additional description of Figure S6.

Row cluster A contained EPeps present in single hexa- and tetraploid samples. The EPeps of row cluster B were present in the majority of di- and tetraploid samples and some hexaploid samples. The EPeps of row cluster C were present in all samples and contained only the epitope PFY. Row cluster D contained EPeps only present in hexaploid samples. The EPeps of row cluster E were present in all hexaploid samples and some samples of the other species. This cluster included the 33-mer (second last EPep).

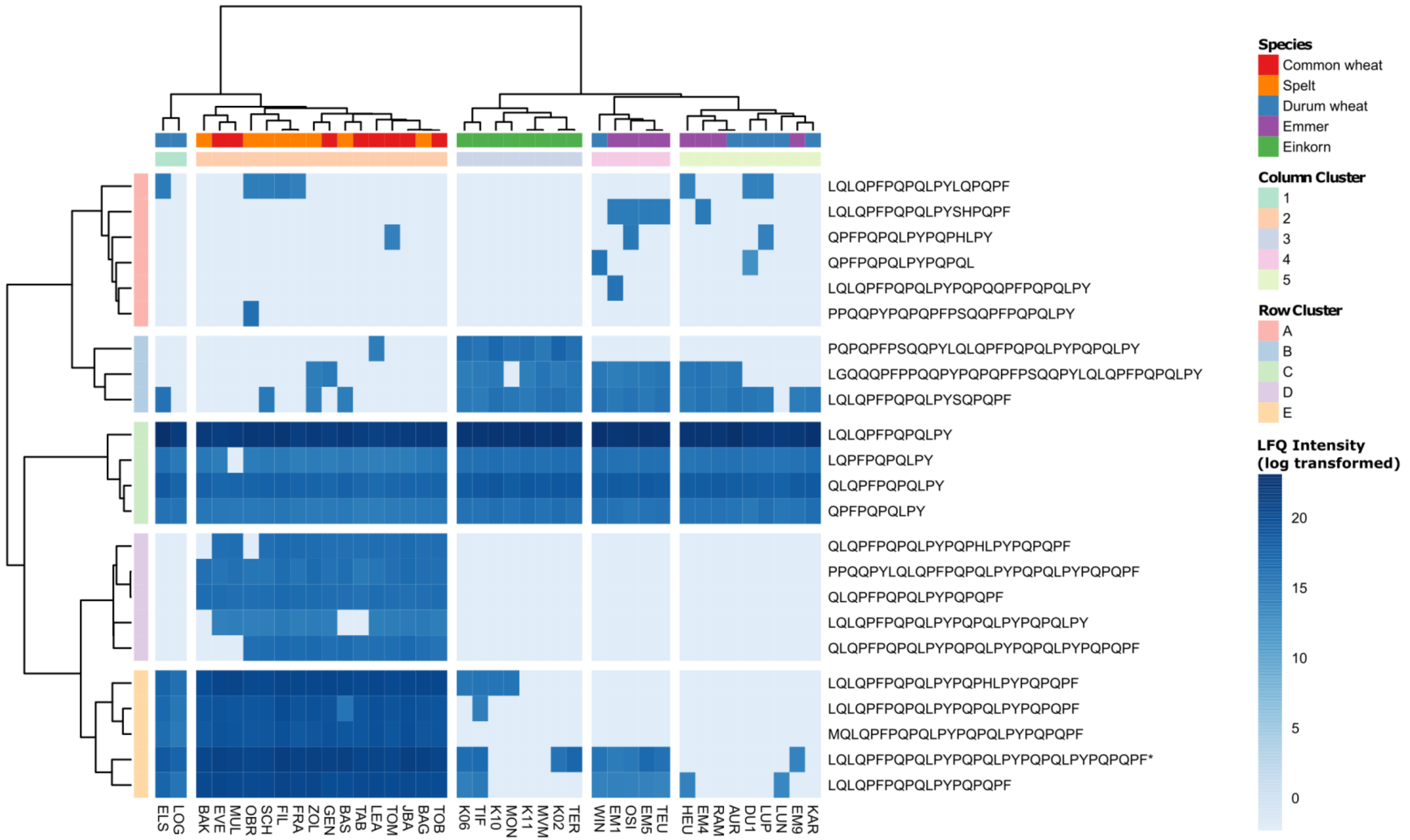

Figure S6. Intensity based on label-free quantitation of identified peptides with celiac disease active epitopes PYPQPQLPY, PFPQPQLPY and PQPQLPYPQ (epitope group 3) belonging to α-gliadin proteins in common wheat, spelt, durum wheat, emmer and einkorn samples, 33-mer is indicated by an asterisk.

Text S7. Additional description of Figure 8.

The EPeps of row cluster A were present mainly in tetraploid samples, additionally in some hexaploid samples and one single diploid sample (K11). Row cluster B contained two EPeps that were present in all hexa- and tetraploid samples and the majority of diploid samples. The EPeps of row cluster C were present in the majority of hexa- and tetraploid samples and one single diploid sample (K02). The EPeps of row cluster D were present in single samples of all species. Row cluster E contained EPeps present in hexa- and diploid samples and some tetraploid samples. The EPeps of row cluster F were present in the majority of hexaploid samples and some di- and tetraploid samples.

Text S8. Additional description of Figure S7.

The EPeps of row cluster A were present in the majority of the common wheat samples and five emmer samples. Row cluster B contained EPeps present in single samples of all species. Row cluster C contained EPeps mainly present in hexaploid samples and in one durum wheat sample (ELS). The EPeps of row cluster D were present in the hexaploid samples of column cluster 1 and tetraploid samples of column cluster 3. The EPeps of row cluster E were present in all hexa- and tetraploid samples and not in einkorn.

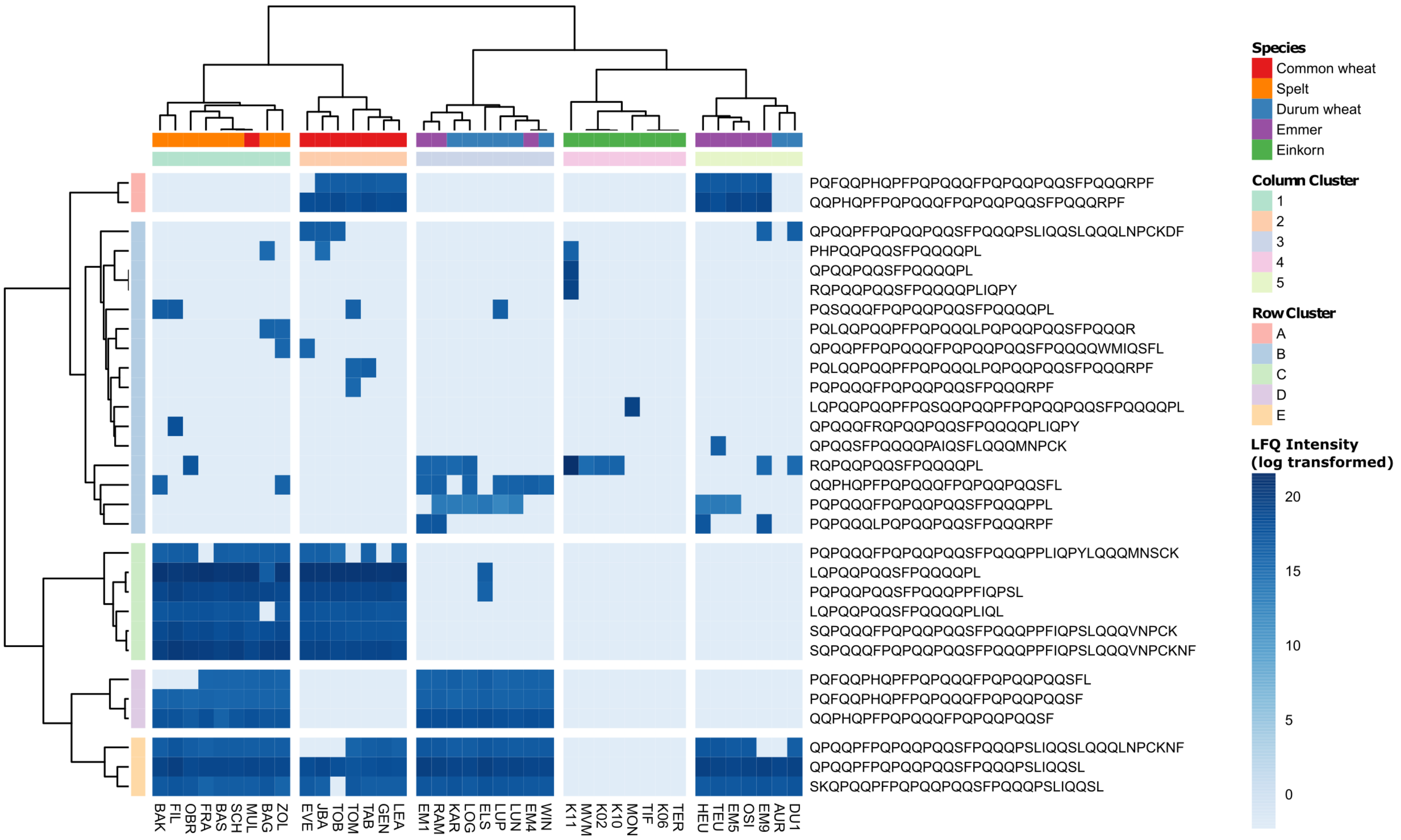

Figure S7. Intensity based on label-free quantitation of identified peptides with celiac disease active epitopes PQQSFPQQQ, SQPQQQFPQ and PQPQQQFPQ (epitope group 4) belonging to γ-gliadin proteins in common wheat, spelt, durum wheat, emmer and einkorn samples.

Text S9. Additional description of Figure S8.

Row cluster A contained EPeps present in some samples of all species. Row cluster B contained EPeps present in the majority of hexaploid samples and single samples of the other species. The EPeps of row cluster C were present in hexa- and tetraploid samples but not in einkorn. The EPeps of row cluster D were present in some hexa- and tetraploid samples but not in einkorn. The EPeps of row cluster E were mainly present in emmer samples, but also in all other species.

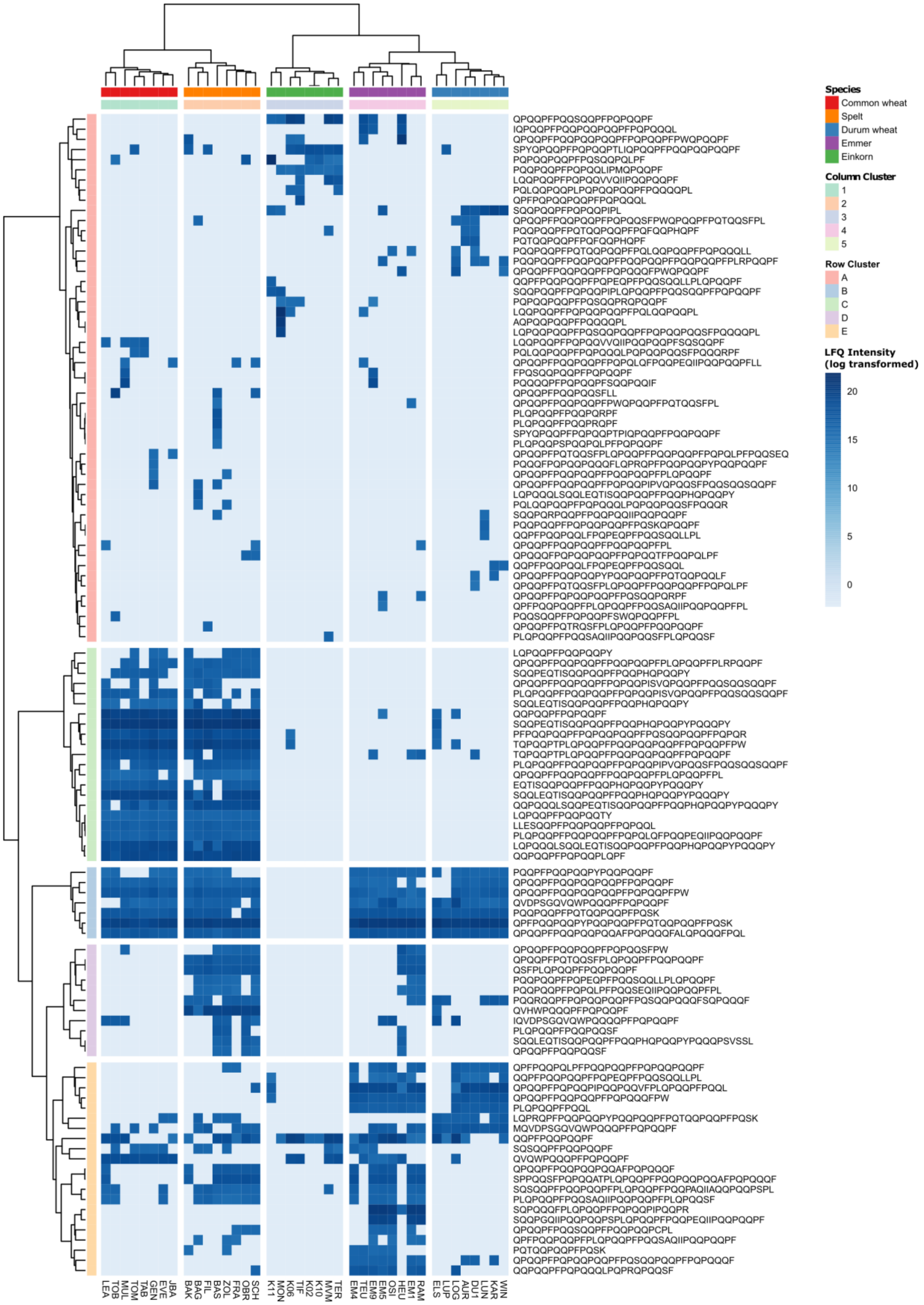

Figure S8. Intensity based on label-free quantitation of identified peptides with celiac disease active epitopes QQPQQPYPQ, QQPQQPFPQ, QQPFPQQPQ, PFPQPQQPF, LQPQQPFPQ and PQPQQPFPW (epitope group 5) belonging to γ- and ω-gliadin proteins in common wheat, spelt, durum wheat, emmer and einkorn samples.
